# iDPF-PseRAAAC: A Web-Server for Identifying the Defensin Peptide Family and Subfamily Using Pseudo Reduced Amino Acid Alphabet Composition

**DOI:** 10.1371/journal.pone.0145541

**Published:** 2015-12-29

**Authors:** Yongchun Zuo, Yang Lv, Zhuying Wei, Lei Yang, Guangpeng Li, Guoliang Fan

**Affiliations:** 1 The Key Laboratory of Mammalian Reproductive Biology and Biotechnology of the Ministry of Education, College of life sciences, Inner Mongolia University, Hohhot, China; 2 College of Bioinformatics Science and Technology, Harbin Medical University, Harbin, China; 3 Laboratory of Theoretical Biophysics, School of Physical Science and Technology, Inner Mongolia University, Hohhot, China; The University of Hong Kong, HONG KONG

## Abstract

Defensins as one of the most abundant classes of antimicrobial peptides are an essential part of the innate immunity that has evolved in most living organisms from lower organisms to humans. To identify specific defensins as interesting antifungal leads, in this study, we constructed a more rigorous benchmark dataset and the **iDPF-PseRAAAC** server was developed to predict the defensin family and subfamily. Using reduced dipeptide compositions were used, the overall accuracy of proposed method increased to 95.10% for the defensin family, and 98.39% for the vertebrate subfamily, which is higher than the accuracy from other methods. The jackknife test shows that more than 4% improvement was obtained comparing with the previous method. A free online server was further established for the convenience of most experimental scientists at http://wlxy.imu.edu.cn/college/biostation/fuwu/iDPF-PseRAAAC/index.asp. A friendly guide is provided to describe how to use the web server. We anticipate that **iDPF-PseRAAAC** may become a useful high-throughput tool for both basic research and drug design.

## Introduction

Defensins are important small, basic, cysteine-rich, antimicrobial, and cationic peptides that are abundant and widely distributed [1, 2]. Defensins are widely distributed in multiple tissues in the body, most notably leukocytes and epithelial surfaces. They are often present at high concentrations[3, 4] and play an essential role in the innate immunity of their hosts from insects and plants to amphibians and mammals[5]. Membrane permeabilization is the crucial step in defensin-mediated antimicrobial activity and cytotoxicity[3, 6]. Defensins from different origins exhibit structural and functional similarities with phylogenetic relationships between different types of defensins. Mature defensins amino acids sequences are highly variable in each defensin family and subfamily [7–9]. Accurately identifying the types of defensins will be helpful in analyzing their specificitis for various microbial targets, provide novel insights for understanding their function, and facilitate antimicrobial drugs targets discovery.

Biochemical experimental methods are highly reliable for elucidating types of defensins, such as nuclear magnetic resonance (NMR) spectroscopy [10]. However, such the experimental techniques are time-consuming and expensive. Bioinformatics methods can timely provide useful information and insights for both basic research and antibiotics design[11, 12]. Thus, better understanding the distinct functions of defensin proteins requires an automated method for timely and reliably annotating the families of many defensin proteins. In our previous study, four defensin families (vertebrate, plant, insect and other defensins) were successfully classified using the increment of diversity (ID) method [13]. In another work the authors developed the DEFENSINPRED classifier to predict human defensin proteins and their types based on pseudo amino acid compositions[14].

However, further work is necessary because the datasets constructed in those methods were too small to reflect a statistical profile and did not impose a rigorous cutoff threshold[15, 16] to exclude the redundant samples in the existing defensin datasets. Moreover, a better web-server for defensins is also needed. In the present work, we constructed a more rigorous benchmark dataset to train the program, and a support vector machine (SVM) classifier was further proposed to classify these five defensin families. An 4% improvement was obtained compared with the previous method. For the convenience of experimental scientists, a free online server **iDPF-PseRAAAC** was first established. A friendly guide was further provided to describe how to use the web server.

## Materials and Methods

### Dataset

**Fig 1:**
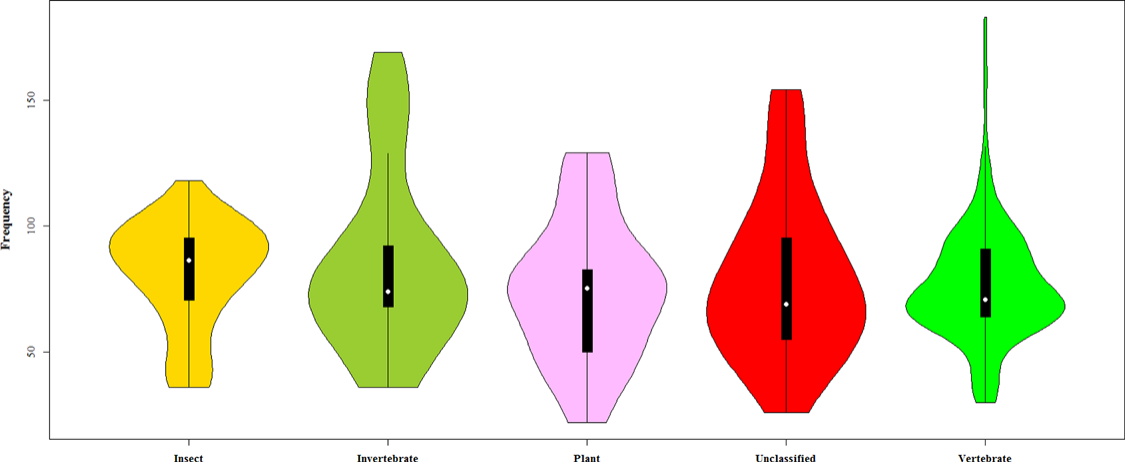
Violin plots show the length distribution of five defensin families.

With rapidly increasing interest in defensins, the Defensins Knowledgebase is available, which is a manually curated database and information source devoted to the defensin family of antimicrobial peptides [17]. The benchmark data set S for the defensin proteins in this study was taken from the Defensins Knowledgebase, which currently contains more than 500 defensin sequences ranging from prokaryotes to eukaryotes. To prepare a high-quality dataset, the program CD-HIT[18] was used to remove the defensin proteins with ≥ 80% pairwise sequence identity to any other protein. Highly similar data will surely lead to overestimation of the performance of the proposed methods. If the sequence identity cutoff is set to a lower percentage (such as 25%), the results will be more objective and reliable. However, in this study we did not use such a stringent criterion because the currently available data do not allow us to do so. The proposed method is a sequence-dependent predictor, the input feature vectors are only derived from the primary amino acids sequence. So there is needed enough amino acids and dipeptide compositions to train the multi-classifier module. For the defensin peptides are polypeptides of fewer than 100 amino acids (See Fig 1) [3]. Besides, for ensure the data reliable, this dataset is a manually curated database. All of the families annotation are gathered from bibliographic databases and sequence databases literature sources. If the sequence identity cutoff is set to a lower percentage (such as 25%), the numbers of proteins for family subsets would have been too few to have statistical significance. And the imbalanced data cause classifiers to tend to overfit and to perform poorly in particular on the minority class[19, 20]. Finally, we obtained a dataset S composed of 333 defensin proteins classified into five families, as formulated by the following equation:

$$S=S_{1}\cup S_{2}\cup S_{3}\cup S_{4}\cup S_{5}$$ (1)

where the subset $S_{1}$ contains 60 insect defensins, $S_{2}$ contains 34 invertebrate defensins, $S_{3}$ contains 42 plant defensins, $S_{4}$ contains 40 unclassified defensins and $S_{5}$ contains 157 vertebrate defensins, and ∪ represents the symbol for “union” in set theory. The length distribution of the five families is depicted in Fig 1. For the readers’ convenience, the 333 defensin proteins sequences and codes are in S1 File.

### Reduced Amino Acid Alphabet

In this study, the reduced amino acid alphabet composition(RAAAC) clustered by Protein Blocks(PBs) are used to predict defensins family and subfamily[21, 22], which is composed of 16 average protein fragments of 5 residues in length. The Protein Blocks have proven their efficiency both in description and prediction of longer fragments[23–25]. Once the databank was encoded in terms of PBs, sequence specificity was computed. Each PB was so associated with a set of enlarged sequence windows [–w; +w] of length l. An amino acid occurrence matrix of dimension 20 × l was computed for each PB. Then, each matrix was transformed into propensities matrix. Finally, all the matrices were compiled to create a matrix F of size 20 ×m with m, a vector of length 16l. The distance between two kinds of amino acids *i* and *j* was computed by using the D(aa*i*, aa*j*). Then a hierarchical clustering using all the amino acid occurrence matrices of the 16 PBs was performed, each resulting amino acid cluster represents amino acids that showed the same over- and under-representations upon all the PBs.

Different defensin peptides usually have specific functional regions, such as β-sheet-rich fold and framework of six disulphide-linked cysteines. Based on the similarity of their functional and physicochemical features in proteins, the 20 amino acids can be clustered into some smaller groups [26, 27]. The reduced amino acids not only can simplify the complexity of the protein system, but also improve the ability in finding structurally conserved regions and the structural similarity of entire proteins[13, 28]. The reduced amino acid alphabet derived from Protein Blocks method has the ability for abstracting useful functional and conservative feature. And it also is helpful for simplifying the amino acids composition of defensin peptide and improving the ability in finding structurally conserved regions and the structural similarity of entire proteins.

Up to now, the Protein Blocks method has successful been used to analyze long protein fragments and to predict functional regions[21, 22], and the results have proven their efficiency both in description and prediction of longer fragments, such as protein structure mining[23, 24], outer membrane proteins analysis[23] and backbone structure prediction of proteins[25]. Our previous researches have also demonstrated that this feature selection method will be useful for analyzing the conservative domain and understanding function evolution of defensin protein [13, 29].

### Support Vector Machine (SVM)

SVM is a powerful and popular method for pattern recognition that has been widely used in biology classification based on statistical learning theory[30–32]. In training an SVM classification system, proteins are represented by sequence-derived properties and are projected onto a hyperspace where the proteins in a family are separated from proteins outside the family by a hyperplane. By projecting a new sequence onto this hyperspace, the SVM system can determine whether or not the corresponding protein belongs to the family based on its location with respect to the hyperplane [33].

In the current study, the LIBSVM 3.0 package was used to implement of SVM[34]; it can be downloaded for free from the website (http://www.csie.ntu.edu.tw/_cjlin/libsvm). Four types of kernel functions, a linear function, polynomial function, sigmoid function and radial basis function (RBF), can be used for predictions in this software. Empirical studies show demonstrated that the RBF outperforms the remaining three types of kernel functions in nonlinear classification[31]. Thus, the RBF kernel function was used in the current work. All the computations were performed using LIBSVM-3.0 standard package (Chang and Lin, 2001). The various user-defined parameters, e.g., kernel parameter γ and regularization parameter C were optimized on the training dataset. The predictor obtained via the aforementioned procedure is called **iDPF-PseRAAAC**, where “i” stands for “identify”, “DPF” for “defensin peptide family”, “Pse” for “pseudo”, “R” for “”reduced”, “AAA” for “amino acid alphabet”, and “C” for “composition”.

### Multi-class SVM

Prediction of defensin family classes is a multi-classification problem. SVM is regarded as a typical binary classifier[35, 36]. The methods of applying SVM to solve multi-class classification problems have one-against-one(OAO), one-against-all(OAA) and directed acyclic graph SVM (DAGSVM)[37]. In the present study, we adopt the “One-against-one” approach to transfer it into a two-class problem. This method involves construction of individual binary SVM classifier corresponding to each pair of the classes. Hence, if there are K classes, OAO will construct a total of K(K-1)/2 classifiers. Each classifier plays a role in classifying of one class and another class. Classifier *i j*, named *f*_*ij*_, is trained using all the patterns from class *i* as positive instances, all the patterns from class *j* as negative instances, and disregarding the rest. The classifiers then are combined using majority voting scheme. Predictions are made with each binary classifiers and label is assigned to a class with maximum number of votes. In case when tie arise, i.e. two classes have identical votes, label assignment to the class is made on the basis of smallest index. More details for one-against-one(OAO) of SVM classification can be found in can be found in[37].

### Performance Evaluation

This method's performance was measured based on sensitivity (Sn), specificity (Sp), Matthew’s correlation coefficient (MCC) and overall accuracy (OA), which were defined as follows:

$$\{\begin{array}{l} \;\text{Sn}(i)=\frac{\text{TP}(i)}{\text{TP}(i)+\text{FN}(i)}\\ \;\text{Sp}(i)=\frac{\text{TN}(i)}{\text{TN}(i)+\text{FP}(i)}\\ \;\text{MCC}(i)=\frac{\text{TP}(i)\times \text{TN}(i)-\text{FP}(i)\times \text{FN}(i)}{\sqrt{\left[\text{TP}(i)+\text{FP}(i)][\text{TP}(i)+\text{FN}(i)][\text{TN}(i)+\text{FP}(i)][\text{TN}(i)+\text{FN}(i)\right]}}\\ \;\text{OA}=\frac{1}{N}\sum_{i=1}^{M}\text{TP}(i) \end{array}$$ (2)

where TP(*i*), TN(*i*), FP(*i*), and FN(*i*) represent true positive, true negative, false positive and false negative of family *i*; *M* = 5 is the number of subsets while *N* the number of the total samples in S.

## Results and Discussion

### Cross Validation

Three cross-validation methods, namely the sub-sampling (or K-fold cross-validation) test, independent dataset test and jackknife test, are often used to evaluate the quality of a predictor[38]. Among the three methods, the jackknife test is the least arbitrary and most objective as demonstrated in[39] and can always yield a unique result for a given benchmark dataset hence. The jackknife test has been widely recognized and increasingly adopted by investigators to examine the quality of various predictors[40–44]. Accordingly, the jackknife test was used to examine the performance of the model proposed in the current study.

### Defensin Family Prediction

**Fig 2:**
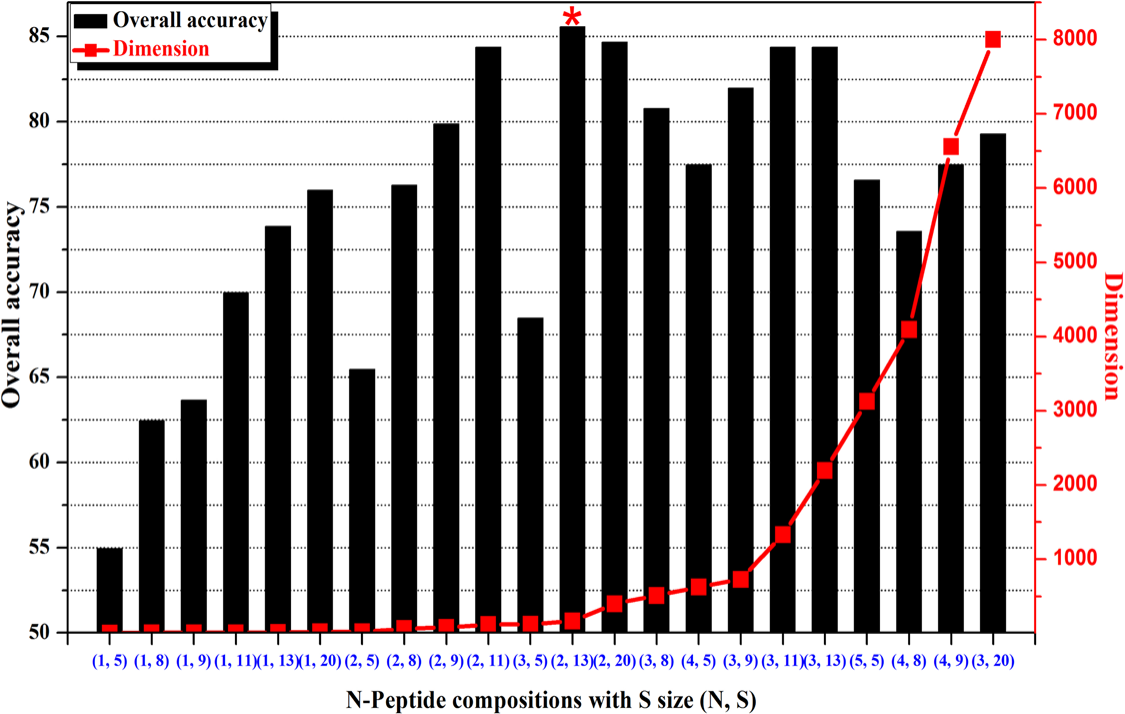
The predictive overall accuracy of defensins families based on different *N*-peptide composition with S size alphabet (N, S).

The jackknife results obtained using the **iDPF-PseRAAAC** and the benchmark dataset S based on different sizes(S) and N-peptide compositions(N) are depicted in Fig 2. Fig 2 shows the prediction results for the overall accuracy of the defensin families based on N-peptide composition with S size alphabet (N, S). As the dimensions increases, N-peptides provide progressively more detailed sequential information. However, the predictive ability did not increased linearly with dimension increase; for example, when the tripeptides composition (3, 20), 8000 dimensions, was selected as the input parameter, the overall accuracy for predicting five defensins families was only 79.28%. The results reflect the notion that a larger dimension does not necessarily result in better performance, and the prediction ability is not always better when the feature dimensions increase. Excessively large dimensions typically lead to information redundancy or noise, which results in bad prediction accuracy.

**Fig 3:**
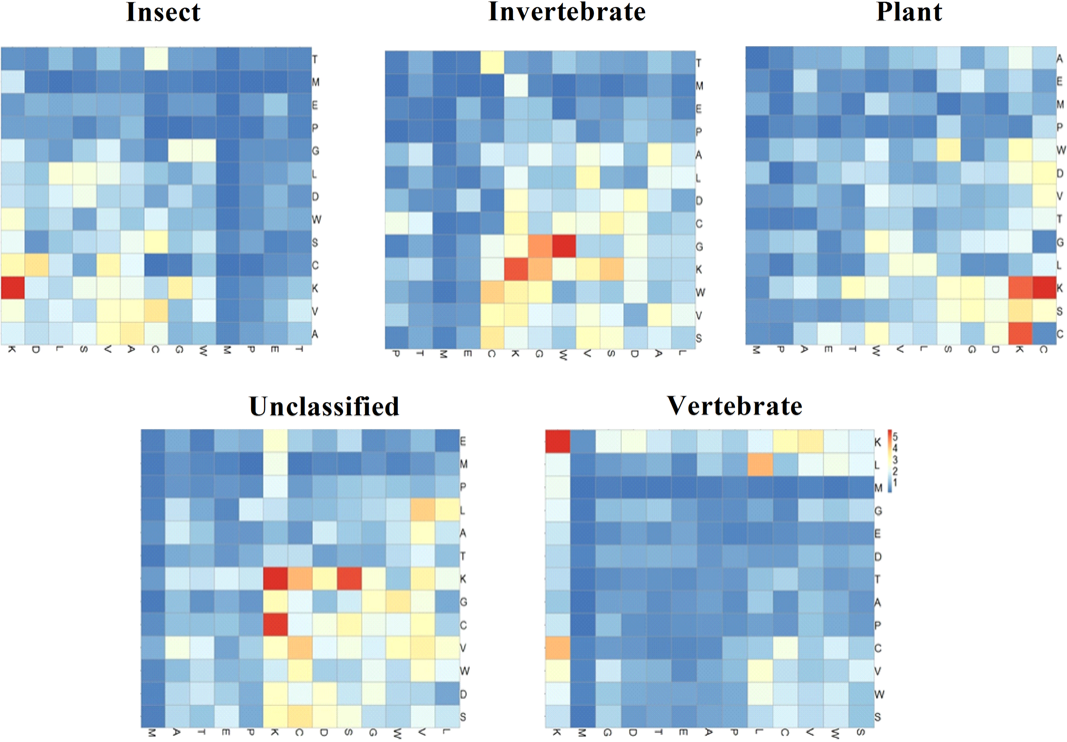
The heatmap shows the adjacent correlation of 13 reduced amino acids for five different defensin families.

The Fig 3 heatmap shows the adjacent correlation of 13 reduced amino acids for five different defensin families. From the prediction performance based on different vector dimensions depicted in Fig 2, we observed that the overall accuracy reached a maximum 85.59% based on 2-peptide composition of 13 reduced amino acids (N = 2, S = 13). Table 1 shows the Jackknife results obtained using **iDPF-PseRAAAC** to identify defensin family with dipeptide (N = 2) composition based on different reduced amino acid alphabet approaches. As shown in Table 2, 2-peptide compositions with alphabet of 13 (N, S) outperformed the other reduced amino acid alphabet sizes. The largest defensin family, vertebrate defensins, yielded the best success rate at 99.36%. The 10-fold cross-validation has been performed to examine the comparability of our method. The prediction results are similar to the jackknife test (Total accuracy: 83.78% Vs 85.59%, S1 Table).

**Table 1:** Results obtained by iDPF-PseRAAAC in identifying defensin peptide families with dipeptide case(N = 2).

			*N*-Peptide compositions of RAAA with S size (N, S)					
Family	Subset	Metrics	(2, 20)	(2, 13)	(2, 11)	(2, 9)	(2, 8)	(2, 5)
			400	**169**	121	81	64	25
Insect	$S_{1}$	Sn(%)	88.33	**90.00**	83.33	76.67	81.67	58.33
		Sp(%)	98.53	**97.07**	96.34	95.60	96.70	91.94
		MCC	0.89	**0.86**	0.80	0.73	0.79	0.51
Invertebrate	$S_{2}$	Sn(%)	64.71	**61.76**	61.76	64.71	55.88	38.24
		Sp(%)	96.32	**97.32**	96.99	95.32	97.66	95.99
		MCC	0.62	**0.64**	0.62	0.59	0.60	0.39
Plant	$S_{3}$	Sn(%)	83.33	**90.48**	90.48	80.95	64.29	57.14
		Sp(%)	99.66	**98.97**	97.94	98.63	98.63	97.25
		MCC	0.89	**0.90**	0.87	0.83	0.72	0.61
Unclassified	$S_{4}$	Sn(%)	45.00	**40.00**	47.50	42.50	17.50	17.50
		Sp(%)	96.59	**96.93**	95.90	95.90	96.93	95.90
		MCC	0.49	**0.46**	0.49	0.44	0.22	0.19
Vertebrate	$S_{5}$	Sn(%)	98.09	**99.36**	97.45	93.63	96.82	88.54
		Sp(%)	85.80	**88.64**	91.48	85.80	71.59	65.34
		MCC	0.84	**0.88**	0.89	0.79	0.70	0.55
	OA(%)		84.68	**85.59**	84.38	79.88	76.28	65.47

The bold values show the best results.

**Table 2:** The prediction results for vertebrate subfamilies based on 2-peptide composition of 13 reduced amino acids (N = 2, S = 13).

Subfamily	Alpha-type	Beta-type	Theta-type	Sn(%)	Sp(%)	MCC
Alpha-type	69	3	0	95.83	100	0.97
Beta-type	0	172	0	100	94.81	0.96
Theta-type	0	1	4	80	100	0.89
Overall accuracy(%)	98.39					

For further comparison, the amino acid (i.e., N = 1) and tripeptide (i.e., N = 3) results were also calculated and were in S2 Table, which shows that none of the results exhibit a higher success rate than N = 2. The data indicat that the reduced amino acid composition provides a greater weight of compositional bias to proteins with a signal at different sequence regions. Subsequently, the adjacent correlation for 13 reduced amino acids was analyzed and depicted using a heatmap plot.

### Vertebrate Defensin Subfamily Prediction

Vertebrates include three distinct defensin subfamilies, Alpha-, Beta-, and Theta-defensins, which exhibit a broad spectrum of antimicrobial activities against bacteria, fungi, and viruses. Subsequently, the proposed method was used to predict the vertebrate defensins subfamily. The prediction results in Table 2 show that the best overall accuracy was 98.39%, and the Mathew's correlation coefficients (MCC) for the Alpha-type, Beta-type and Theta-type are 0.97, 0.96 and 0.89, respectively. Such high accuracies demonstrate that the proposed method is an effective and powerful approach for predicting defensin subfamilies.

### Comparison with Previous Methods

**Fig 4:**
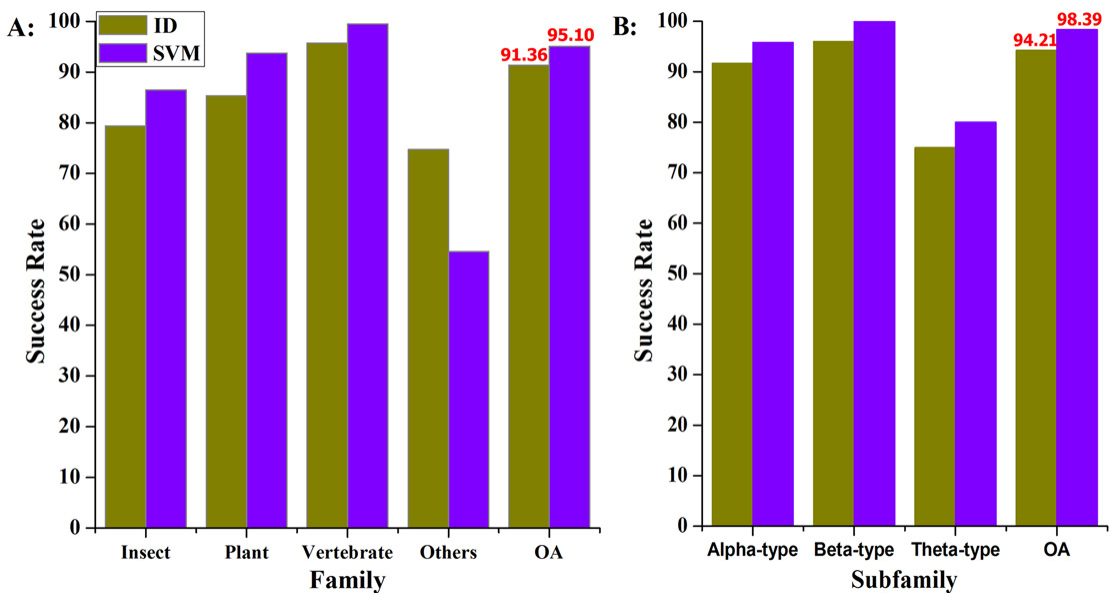
Comparing the performance of the proposed method with our previous methods. **A:** indicated the prediction results of defensin family; **B:** indicated the prediction results of vertebrate defensin subfamily.

To further demonstrate the performance of the proposed method, it is necessary to be compared with other existing methods. However, directly comparing the results is not objective and strict examination due to the different benchmark datasets used. Therefore, we repeated the feature selection and prediction process on the previous dataset. The jackknife cross-validated accuracies are depicted in Fig 4. Obviously, our proposed method yields the highest predictive success rate.

According to Fig 4, when the 169 reduced dipeptides are used, our method can achieve a maximum overall accuracy (OA) of 95.10% for the defensin family, and 98.39% for the vertebrate subfamily, which is higher than the maximum accuracy obtained using other methods. Although the success rate for others family and subfamily obtained using our method is not better, the accuracy of the other families and subfamilies are dramatically better than using other methods, and Fig 4 clearly indicate that the proposed method is more powerful than our previous method.

Subsequently, it is instructive to compare the overall success rate from **iDPF-PseRAAAC** with the success rate for weighted random guess (WRG)[45]. The overall success rate based on the OA to identify the defensin proteins among their four subfamilies WRG is given by [45].

$$\text{OA}(\text{WRG})=\frac{{\left(N_{1}\right)}^{2}+{\left(N_{2}\right)}^{2}+{\left(N_{3}\right)}^{2}+{\left(N_{4}\right)}^{2}+{\left(N_{5}\right)}^{2}}{N^{2}}$$ (3)

where *N* is the number of defensin proteins in the benchmark dataset S, *N*_1_ the number of defensin proteins in the subset $S_{1}$, *N*_2_ the number of defensin proteins in the subset $S_{2}$, and so forth (see **Eq 1**). Substituting these data into **Eq 3**, we obtain the following.

$$\text{OA}(\text{WRG})=\frac{{\left(60\right)}^{2}+{\left(34\right)}^{2}+{\left(42\right)}^{2}+{\left(40\right)}^{2}+{\left(157\right)}^{2}}{333^{2}}=29.55\%$$ (4)

In contrast, the best overall success rate for **iDPF-PseRAAAC** was 85.59%. Compared with the results in Eq 4, the overall success rate for **iDPF-PseRAAAC** is approximately 56% higher than using a weighted random guess, which indicate that **iDPF-PseRAAAC** may be an easy and useful tool for timely identifying defensin proteins families.

### Web-Server Guide

For the convenience of most experimental scientists, below, we provide a step-by-step guide on how to use the **iDPF-PseRAAAC** web-server to achieve the desired results.

**Fig 5:**
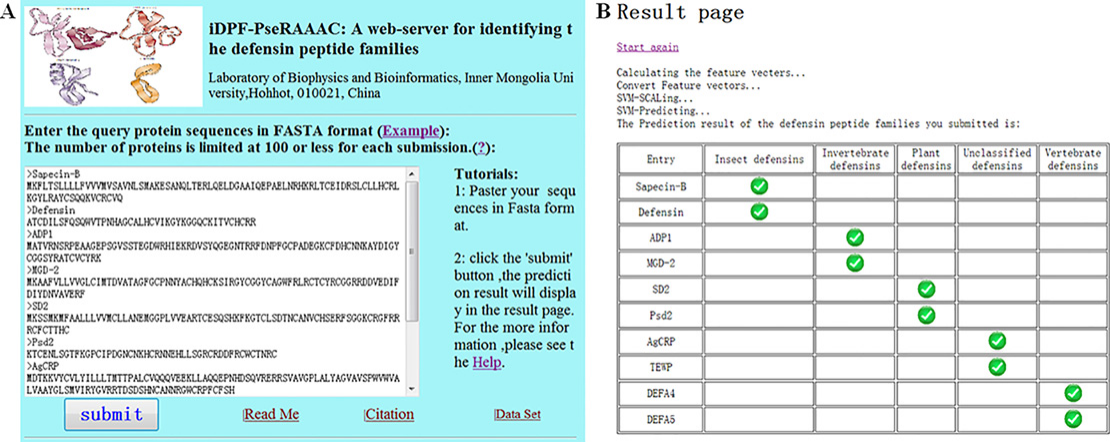
A semi-screenshot to show the top page of the iDPF-PseRAAAC web-server.

**Step 1.** Open the web server at http://wlxy.imu.edu.cn/college/biostation/fuwu/iDPF-PseRAAAC/index.asp and you will see the top page for **iDPF-PseRAAAC** on your computer screen, as shown in Fig 5. Click on the 'Read Me' button to see a brief introduction about the predictor and the caveat for using it.

**Step 2**. Either type or copy/paste the query defensin peptide sequence into the input box at the center of Fig 5. The input sequence should be in the FASTA format. A sequence in the FASTA format consists of a single initial line beginning with a greater than symbol (“>”) in the first column, followed by lines of sequence data. The words immediately following the “>” symbol in the single initial line are optional and only used for the identification and description. The sequence ends if another line starting with a “>” appears; this indicates the start of another sequence. Example sequences in FASTA format can be viewed by clicking on the 'Example' button right above the input box.

**Step 3**. Click on the 'Submit' button to see the predicted result. For example, if you use the query defensin protein sequences in the 'Example' window as the input, after clicking the 'Submit' button, you will see the "Result page" shown on the screen of your computer. All these results are fully consistent with the experimental observations. It takes approximately a few seconds for the above computation before the predicted result appears on your computer screen; for query sequences and longer each sequences, more time is typically required.

**Step 4**. Click on the 'Citation' button to find the relevant papers that document the detailed development and algorithm of **iDPF-PseRAAAC**.

**Step 5**. Click on the 'Data' button to download the benchmark datasets used to train and test the **iDPF-PseRAAAC** predictor.

## Conclusions

Defensins also play important regulatory roles in the immune systems of animals and plants, acting as a bridge between innate and adaptive immunity in vertebrates. In this study, a promising method, **iDPF-PseRAAAC,** was developed to improve prediction performance for defensin proteins. The use of reduced amino acid alphabets not only provides an efficient and accurate means of protein vectorization for sequence-based protein classification systems but also remarkably improves computational efficiency. High predictive accuracies demonstrate that our proposed method is a potentially useful tool for classifying defensin family.

## Supporting Information

S1 File The 333 defensin proteins sequences and codes.docx. (DOCX)

S1 Table The prediction results of 10-fold cross-validation for the benchmark dataset. (DOCX)

S2 Table The results obtained by iDPF-PseRAAAC in identifying defensin peptide families with (A) single amino acid case (N = 1) and (B) tripeptide case (N = 3). (DOCX)
