# Supplementary material for: iDPF-PseRAAAC: A Web-Server for Identifying the Defensin Peptide Family and Subfamily Using Pseudo Reduced Amino Acid Alphabet Composition
Source: PLoS One. 2015 Dec 29;10(12):e0145541. doi: 10.1371/journal.pone.0145541 (PMC4694767; doi:10.1371/journal.pone.0145541)
Supplement: S1 File — (DOCX) [file pone.0145541.s001.docx]

**Insect_60**

>Sapecin-B

MKFLTSLLLLFVVVMVSAVNLSMAKESANQLTERLQELDGAAIQEPAELNRHKRLTCEIDRSLCLLHCRLKGYLRAYCSQQKVCRCVQ

>Defensin

ATCDILSFQSQWVTPNHAGCALHCVIKGYKGGQCKITVCHCRR

>Phormicin

MKFFMVFVVTFCLAVCFVSQSLAIPADAANDAHFVDGVQALKEIEPELHGRYKRATCDLLSGTGINHSACAAHCLLRGNRGGYCNGKGVCVCRN

>Defensin

MRTFLVTFVLVVVVGVISAYPSNPVEVEAEDFDAQDPDLQTFQDTFYEVPQVHSRQKRATCDLLSAFGVGHAACAAHCIGHGYRGGYCNSKAVCTCRR

>Defensin

ATCDALSFSSKWLTVNHSACAIHCLTKGYKGGRCVNTICNCRN

>Defensin

MSRFIVFAFIVAMCIAHSLAAPAPEALEASVIRQKRLTCDLLSFEAKGFAANHSLCAAHCLAIGRKGGACQNGVCVCRR

>Defensin

MKFFVLVAIAFALLACVAQAQPVSDVDPIPEDHVLVHEDAHQEVLQHSRQKRATCDLLSKWNWNHTACAGHCIAKGFKGGYCNDKAVCVCRN

>Royalisin

MKIYFIVGLLFMAMVAIMAAPVEDEFEPLEHFENEERADRHRRVTCDLLSFKGQVNDSACAANCLSLGKAGGHCEKGVCICRKTSFKDLWDKYFG

>DEF1

MKCATIVCTIAVVLAATLLNGSVQAAPQEEAALSGGANLNTLLDELPEETHHAALENYRAKRATCDLASGFGVGSSLCAAHCIARRYRGGYCNSKAVCVCRN

>Defensin

GFGCPLDQMQCHRHCQTITGRSGGYCSGPLKLTCTCYR

>DEFC

MRTLTVVCFVALCLSAIFTTGNALPGELADDVRPYANSLFDELPEESYQAAVENFRLKRATCDLLSGFGVGDSACAAHCIARRNRGGYCNAKKVCVCRN

>DEFB

MKSITVICFLALCTVAITSAYPQEPVLADEARPFANSLFDELPEETYQAAVENFRLKRATCDLLSGFGVGDSACAAHCIARGNRGGYCNSQKVCVCRN

>Defensin

FTCDVLGFEIAGTKLNSAACGAHCLALGRTGGYCNSKSVCVCR

>SMD2

MKFFSLFPVIVVVVACLTMRANAAPSAGNEVDHHPDYVDGVEALRQLEPELHGRYKRATCDLLSMWNVNHSACAAHCLLLGKSGGRCNDDAVCVCRK

>SMD1

MKFLNVVAIALLVVACLAVYSNAAPHEGVKEVAAAKPMGITCDLLSLWKVGHAACAAHCLVLGDVGGYCTKEGLCVCKE

>Sapecin

MKSFIVLAVTLCLAAFFMGQSVASPAAAAEESKFVDGLHALKTIEPELHGRYKRATCDLLSGTGINHSACAAHCLLRGNRGGYCNGKAVCVCRN

>Termicin

ACNFQSCWATCQAQHSIYFRRAFCDRSQCKCVFVRG

>DEF2

MKSFIAAAVIALICAIAVSGTTVTLQSTCKLFTADVVSSITCKMYCVIKGKTGGYCNSEGLCTCRAEDLHFLLKPIINKD

>DEF3

MKFAVVSFLLVALLGLVAVGEAQLKNLACVTNEGPKWANTYCAAVCHMSGRGAGSCNAKDECVCSMT

>DEF4

MRTIAQLVTLFGAIALLLLVSTEMTFANPLSPNSPAERPHIQPFQMASAPLVAQSRSAMVQTLTCTNPTCSAQCRGRGYRRGSCTIGRCFCSYV

>Holotricin-1

VTCDLLSLQIKGIAINDSACAAHCLAMRRKGGSCKQGVCVCRN

>Defensin1

MKFFITFTFVLSLVVLTVYSAPREFAEPEEQDEGHFRVKRFTCDVLSVEAKGVKLNHAACGIHCLFRRRTGGYCNKKRVCICR

>GmDefA

MKFYLVLAFLTLCAVAVTALPAGDETRIDLETLEEDLRLVDGAQVTGELKRDKRVTCNIGEWVCVAHCNSKSKKSGYCSRGVCYCTN

>Tenecin-1

MKLTIFALVACFFILQIAAFPLEEAATAEEIEQGEHIRVKRVTCDILSVEAKGVKLNDAACAAHCLFRGRSGGYCNGKRVCVCR

>DefensinA

LTCDILGSTPACAAHCIARGYRGGWCDGQSVCNCRR

>Defensin2

MKFFVLFAILIAIVHASCASVPKVVYDGPIYELRQIEEENIEPDTELMDSNEPLLPLRHRRVTCDVLSWQSKWLSINHSACAIRCLAQRRKGGSCRNGVCICRK

>MdesDEF-1

MNFKLLNLFAMVLFGVVVISNAAKPPSGFLTPDADNDENGNGVEEQTLERFTCDIWQNQAACAIHCIANGFRGGYCNAQKVCVCRR

>Eristalin

MRASIAITFLFGLALCVVCSAVPVVDPEAAAELEFVGEHDLEAVADGTGLRQKRATCDLLSFLNVKDAACAAHCLAKGYRGGYCDGRKVCNCRR

>Galiomicin

MAKNFQSVLLLVCLSFLVIVSSPQNAVQADTLIGSCVWGATNYTSDCNAECKRRGYKGGHCGSFLNVNCWCE

>DFP-6

MKTCLVFAFFLVAVFAAVQAEENDSPQTLPRRLTVRAAQSFGRCNQKQCDADCVKKGYFGGLCTLTSCFCTGSRS

>Defensin

MKNYVFALLVVTAVAIALPNEDKNAPMRVHLLPQKEDESLKLEVTPVKEHHRTRRFTCDLLSGAGVDHSACAAHCILRGKTGGRCNSDRVCVCRA

>Defensin

MVKVYFLVALLFVAVAAIMAAPVEEEYELLEQAGIEERADRQRRVTCDLLSIKGVAEHSACAANCLSMGKAGGRCENGVCLCRKTNFKDLWDKRFG

>Dp1

MKTLAILLVFLVVACVFTAQHPADADCNTKACWALCQREHGIYFRRAVCEGSRCKCILVNGR

>Nc1

MKTLAILLCFLVVVCVFISQHPADAACEFQSCWVTCQRKYNIYFRKAYCEKSKCICVYNYGG

>Nc2

MKTLAILLCFLVVVCVFISQHPADAACDFNSCWATCKAQNGIYFRRAFCDGPTCLCVFLNAG

>Ne2

MKTLAFLLCFLVVVCVFIAQHPADAACNFQSCWAICKAHYGIYFRRAYCDGPNCQCVHLIQG

>Nl

MKTLAILLCFLVVVSVFIAQHPADAACDFQSCWVTCQRQHSIYFIRAFCDGSRCMCVYNNGG

>DefensinA

MKCILSLVTLFLVAVLVHSHPAEWNTHQQLDDALWEPAGEVTEEHVARLKRATCDLFSFRSKWVTPNHAACAAHCLLRGNRGGRCKGTICHCRK

>DefensinC

MKCILSLFTLFLVATLVYSYPAEWNSQHQLDDAQWEPAGELTEEHLSRMKRATCDLLSLTSKWFTPNHAGCAAHCIFLGNRGGRCVGTVCHCRK

>Def1

MKCALSLVTLFLVAALAYSYPADLAQQPLDETEWEQPAGEITEEHGARLKRATCDLFSFESKWFTPNHAACAAHCILLGNRGGHCVGTVCHCRK

>Defensin

MLCLADIRIVASCSAAIKSGYGQQPWLAHVAGPYANSLFDDVPADSYHAAVEYLRLIPASCYLLDGYAAGRDDCRAHCIAPRNRRLYCASYQVCVCRY

>Defensin-1

MKFIVIFVISLCVFAITAYPLDQVEEQDEHQVAHIRVRRVTCDLLSAEAKGVKVNHAACAAHCLLKRKRGGYCNKRRICVCRN

>Defensin-2

MKLLIVALVALFCIFETTAFPTDGEHIRVKRFTCDVLSAEGSFRGVSVKLNHSACATHCLFLKKRGGYCNNKAICVCRN

>Defensin-3

MKLIIIALIALFCVFETTAFPADGEHVRVKRFTCDVLSAEGGFRGVSIKLNHAACAAHCLYLKKRGGYCNDKAVCVCRK

>Defensin

MKTSVALVLLACLLVSAVMTAPLEGPEGRALRHRRVTCDLLSFSSKIFSFNHSACAAHCLAKRKKGGRCVNGVCRCRN

>DEFE

MKSITTLCLAVVCFIALLSVGAAAPQESVSIQDAEHPAEFDTDIQKVQHDQARQIPVEFQRRKRISCDLLSGLGWGHSICAGHCLAISWRYRGGYCNDQGVCVCRT

>Def

ASCDLFSFSSQWVTPNDSVCAAHCLVKGYKGGSCKNKICHCRDKF

>Def

ATCDLLSIFNVNHAACAAHCLAIGRRGGYCNSKQVCVCR

>Def

MKYFTIVAVFLAVAVCYISQSSASPAPNEEANFVHGADALKQLEPELHGRYKRATCDLLSGTGVGHSACAAHCLLRGNRGGYCNGKGVCVCRN

>DEFA

MNSLGTACFAVLCLFAIVSTGNGFPQESADQVQYFANTLFDELPEQSYQAAAENLRLKRATCDLLSGLGVNDSACAAHCIARGNRGGYCNSKKVCVCRN

>DEFB

MQSYTVIGFIALCTMTIGTMAYPQKTVLSDNVQSYANSLFDEIPEESYTNTAAIEHNRQKRATCDLLSGFGVNDSACAVHCILRGNRGGYCNSKKVCVCRN

>Defensin

MVKVVLFVFLIVLAVGAYCAPVDEEFQDDLIEGPVRVKRATCDLLSFEIKGFKLNDSACAAHCIQLGKRGGHCNNSKVCVCRR

>Coprisin

MAKLIAFALVASLCLSMVLCNPLPEEVQEEGLVRQKRVTCDVLSFEAKGIAVNHSACALHCIALRKKGGSCQNGVCVCRN

>LuloDEF

MGIFRVSLCVVVLVAVVAANPAKSSAAKENFDSLQVEDNPSEPQIQPRVTCDLLGPTGWGDALCAAHCISKGYRGGYCNAQKVCVCR

>Defensin

MNGLNLIIIMIVGCCCFVVASGLPSTLNQFPGQKFQIKVSVENGGDDVNYLFDDVKEEIQTNGGRFRRATCDLLSFDTKWGSLNHSACAAHCIALRKGYKGGRCYKQVCRCRK

>NaviDef1-1

MKLLLVVAFIAVAVTAGLSIPLNEFEDLVDFQDWDEAAVDEDAGVRQRRVTCDLLSFGGVVGDSACAANCLSMGKAGGSCNGGICECRKTTFKELWDQRFG

>NaviDef1-2

MKFLIIAVFSAMVVSAALSLPLDELEDLVDVQDWEEAVVDDNAGIRQRRVTCDLLSFGGKVGHSACAANCLSMGKAGGRCNGGVCQCRKTTFGDLWNKRFG

>NaviDef2-2

MKVLVVLAACAVFAGAFGATRIRDGYEDPVFEILGDDIKQDGDNAETVDATDDLSPIKESSDDPTELVQLSYRVRRFSCDVLSFQSKWVSPNHSACAVRCLAQRRKGGKCKNGDCVCR

>Protaetiamycine

MSKFTVLAFIVAMCVMHTFAIPVPEEIEGSIIRQKRVTCDLLSFEILGVALNHSACAAHCLAIGRGGGACQGGICVCRR

>Lw4

MAAVVSSAPPKYEEPIEVLPSEYELSDVNVVEDLQNFLEEALTVEEAEARQARVTCDLLSGIGWNHTFCAAHCIFKGYKGGACNSKGVCVCRR

**Invertebrate_34**

>ADP1

MATVRNSRPEAAGEPSGVSSTEGDWRHIEKRDVSYQGEGNTRRFDNPFGCPADEGKCFDHCNNKAYDIGYCGGSYRATCVCYRK

>MGD-2

MKAAFVLLVVGLCIMTDVATAGFGCPNNYACHQHCKSIRGYCGGYCAGWFRLRCTCYRCGGRRDDVEDIFDIYDNVAVERF

>Cll-dlp

MKAIVVLLILALILCLYAMTTVEGACQFWSCNSSCISRGYRQGYCWGIQYKYCQCQ

>Defensin-A

GFGCPNDYPCHRHCKSIPGRXGGYCGGXHRLRCTCYR

>Varisin

MRGLCICLVFLLVCGLVSATAAAPAESEVAHLRVRRGFGCPLNQGACHNHCRSIRRRGGYCSGIIKQTCTCYRN

>Cg-Def

MKVFVLLTLAVLLMVSADMAFAGFGCPGNQLKCNNHCKSISCRAGYCDAATLWLRCTCTDCNGKK

>Plectasin

MQFTTILSIGITVFGLLNTGAFAAPQPVPEAYAVSDPEAHPDDFAGMDANQLQKRGFGCNGPWDEDDMQCHNHCKSIKGYKGGYCAKGGFVCKCY

>Atesin-3

MQFSTVFLGAVALFSSMAFAVPSENLKVRSSCQLGDIWGAGDAACSASCIAQGEGYHGGHCNDDSVCVCNY

>ADP2

MATQRREISWTFGPLYTWRTTKGYGTTLETTNATSTSSKPSRRYENPYGCPTDEGKCFDRCNDSEFEGGYCGGSYRATCVCYRT

>DefA

MNKLFIVALVVALAVATMAQEVHNDVEEQSVPRVRRGYGCPFNQYQCHSHCSGIRGYKGGYCKGTFKQTCKCY

>AOD

GFGCPWNRYQCHSHCRSIGRLGGYCAGSLRLTCTCYRS

>Longicin

MKVLAVALIFVLVAGLFCTAAAQDDESDVPHVRVRRGFGCPLNQGACHNHCRSIGRRGGYCAGIIKQTCTCYRK

>Hlgut-defensin

MKVFSALFLVGLLLAFLAFAAGDEEDSSKPLVRVRRGFGCPFDERACHAHCQSVGRRGGYCGNFRMTCYCYKN

>Hlsal-defensin

MKLLVVCAALTVLGGLLQGATCDDSDHGFRTAHVDLVCPDNPDNCIQQCVSKGAQGGYCTNEKCTCYEKIPSATKRVRIVA

>Tddefensin

RYCPRNPEACYNYCLRTGRPGGYCGGRSRITCFCFR

>BRAFLDRAFT_96429

MLTYLTYVGCCSSPATRTTVAAQKTLSSIAFLMLSSSRPVSRLRTSVWAAPRFKPKMAPQPRISCDLLSISIGGTNFGDQNSACTAHCLVLGYSGGHCNSHDDNVCACH

>BRAFLDRAFT_122258

MGFFPRKRYKRNTCTSASADDISDSDRNTFRCIMKFLIVAIVLAVLANAEAAPKFKPKMAPQPRITCDLLSFNLPIGSFGDSLCAAHCLALGYRGGYCDDRNASLSNAGGDQCHSPCTLGASQDRVASECCSGIQGSQPADLTLALRGSLSPKDLAPSPRALIGNDLAV

>Atesin-2

MQFTKAIALFSLVATGLAVAAPEDNANANANGNTAPAKPVIGAGEPFDGDFDLLLGGKPNMMLNKRGYGCPNDYACSSYCSSIGRNGGYCGGFLWQTCKCNEKK

>Anisin-1

MQFSAIVLSAVALFGSMTFAAPAPAPDAELMARSSCQLGGIFGAGDAACSASCIRAGTYHGGYCNDKQVCICTH

>Cglosin-1C

MRFILSTLAAGLLATAPLALAQDCSSVVCVLGDGACNRVCEMEGHTEGGKCVPRDGCPAGSEICVCGAKKAKRAVVAADGDELLARALDGVATVDQFVDAVFEKRDGSEAAVDKRSVCCSFPDPVGGLCCEAHCQQIGHLEGGQCTAQNVCVCG

>AorsinC

MRFFGGVIATFVVCSSLADAFCHDSISCMVGGDNVCNNVCVRQGNPNGGRCLPRDGCPGNDICACYPQSKRSDGVIDGDASIREVLKDFGIDGGAEKELNAREKRSISCNFPDPFGGLICENHCAYIGKPGGQCSDQKVCTCN

>Nefisin-2C

MRFTTIVTPLLASATLASALCHNSISCMMGGDSTCNNVCVRQGNPSGGRCLPRDGCPGYDICACYPNSKRSDEVIDGDEPLREALNNMGIDEAAAVKNLDTRDATFDKRSICCSFPDPWGGLCCEDHCSYIGKPGGQCSDKSVCTCN

>AclasinC

MRFALFTPLLAALPLASALCHNSISCALGGDNVCNNVCVRQGNDNGGRCLPRDGCPGYDICACYPRSKRSDEVIDGDAPLREVLKDMGVEDVVNKVDARDVKLDKRSICCSFPDPWGGFCCEDHCSYIGKPGGQCSDKKVCTCN

>Anisin-2

ASCQIGDIWGAGDAACSASCLKDGTYHGGYCNEESVCVCTY

>Defensin-2

MRFTSAIFLVAVIAAFVVMITATGERSEERSEEARASGCKADACKSYCKSLGSGGGYCDQGTWCVCN

>Scapularisin

MRVIAVTLIALLVAGAFMTSSAQEEENQVAHVRVRRGFGCPFDQGACHRHCQSIGRRGGYCAGFIKQTCTCYHN

>DEF1

MKVLAVSLAFLLIAGLISTSLAQNEEGGEKELVRVRRGGYYCPFFQDKCHRHCRSFGRKAGYCGGFLKKTCICVMK

>DEF4

MKVWLVAALIISALGCLAAFPAEGNSQLAHHRVRRGFFCPYNGYCDRHCRKKLRRRGGYCGGRWKLTCICVQ

>Amercin

MKVLAVAFIFVLVAGLVSTADEEDKSQVPLVRVRRGFGCPFNQYQCHSHCLSIGRRGGYCGGSFKTTCTCYN

>ACAJ0367S_Def

MKVICLAVIVLAAADATFGDYYYYDEVPLPGPEGTDPSEIPENPAVHVDESSAENQGIGTVIRDVSYHGKGSQPTEIPETPAVNFDERSAENQLRWKGKRDVSHHGEGNTRRFDNPFGCPIDEGKCFDHCNNKAYDGGYCGGSYRATCICHRK

>AM-383_Def

MKYLAVLVFLLISSTVQVSAQDDDGDDDAALTRVRRGFGCPFNQGACHRHCQSIGRKGGYCSGLFKQTCTCYRH

>AM-1173_Def

MNKSLVIVLVLAIAVAATTAQSVDEPERSHGRVRRGYGCPFNQYECHNHCKGVPGYKGGYCDGFLKMTCRCY

>Defensin

MKFLLLCLVIAFVGMSDAASLQKRVTCDLLSLQIMGNSFGDSACAAHCIGLHHSGGHCSGGVCVCR

>Defensin

MRGLCICLVFILVCGLLTATAAVPAESEAAHLRVRRGGYCSGIIKQTCTCYRN

**Plant_42**

>SD2

MKSSMKMFAALLLVVMCLLANEMGGPLVVEARTCESQSHKFKGTCLSDTNCANVCHSERFSGGKCRGFRRRCFCTTHC

>Psd2

KTCENLSGTFKGPCIPDGNCNKHCRNNEHLLSGRCRDDFRCWCTNRC

>Psd1

KTCEHLADTYRGVCFTNASCDDHCKNKAHLISGTCHNWKCFCTQNC

>LCR78

MASSYTLMLFLCLSIFLIASTEMMAVEGRICERRSKTWTGFCGNTRGCDSQCKRWERASHGACHAQFPGFACFCYFNC

>So-D6

GIFSNMYXRTPAGYFRGPXGYXXN

>So-D5

MFFSSKKCKTVXKTFRGPCVRNAN

>So-D2

GIFSSRKCKTPSKTFKGICTRDSNCDTSCRYEGYPAGDCKGIRRRCMCSKPC

>So-D1

XTCESPSHKFKGPCATNRNCES

>PhD2

MARSICFFAVAILALMLFAAYETEAGTCKAECPTWEGICINKAPCVKCCKAQPEKFTDGHCSKILRRCLCTKPCATEEATATLANEVKTMAEALVEEDMME

>DefensinJ1-2

MAGFSKVIATIFLMMMLVFATGMVAEARTCESQSHRFKGLCFSKSNCGSVCHTEGFNGGHCRGFRRRCFCTRHC

>PhD1

MARSICFFAVAILALMLFAAYDAEAATCKAECPTWDSVCINKKPCVACCKKAKFSDGHCSKILRRCLCTKECVFEKTEATQTETFTKDVNTLAEALLEADMMV

>DefensinJ1-1

MAGFSKVVATIFLMMLLVFATDMMAEAKICEALSGNFKGLCLSSRDCGNVCRREGFTDGSCIGFRLQCFCTKPCA

>RsAFP1

MAKFASIIALLFAALVLFAAFEAPTMVEAQKLCERPSGTWSGVCGNNNACKNQCINLEKARHGSCNYVFPAHKCICYFPC

>AhAMP1

LCNERPSQTWSGNCGNTAHCDKQCQDWEKASHGACHKRENHWKCFCYFNC

>NaD1

MARSLCFMAFAILAMMLFVAYEVQARECKTESNTFPGICITKPPCRKACISEKFTDGHCSKILRRCLCTKPCVFDEKMTKTGAEILAEEAKTLAAALLEEEIMDN

>VrD1

RTCMIKKEGWGKCLIDTTCAHSCKNRGYIGGNCKGMTRTCYCLVNC

>LCR73

MKLSLRFLSVLLLIAFMVLATTAEVSPLDNKICKTRSDRFSGVCISTNNCAIICQQFEHFDGGHCEFDGAFRRCMCTKQC

>LCR59

METVTSLVFIVNLLIIFTSVVNQARGDTCIDGLGYCNNCDERCKAKHGPSSESSCDRSVGVPLCKCYYECESPPSPPAPPKKCDGGAGICSQRCQGQCCDMNCAQKYIGGHGFCNTLGTFSFCQCEYPC

>LCR19

MEKALSLVVFIIFSIMLASVENKVNANTCIEGIGNCQQCDVRCKARHGPAAKGACDSKFQLCTCNYPCGQGPSPPQPKKCYGGAGICSDRCGAQCCNQNCAQKYNQGSGFCDSIGNTSLCKCQYNC

>LCR57

MERTSTSLLFLLSLLIIFASAVNQIRAQTCDENLSSCENCDQRCKAKHGPSSVSKCNGPDGTCGCASFKPAKLCIGATDMCTDKCPTSCCDRQCAIKYKNGKGGCVDYAGYRMCICEYTC

>LCR58

MERITSLVFFASFLIIFVSGVNQTRADSCDESLGLCETCDERCQAKHGPSCISKCDGEVGMLSCTCTYECGPPLPPKGNVCSGGTGMCSGKCPYKCCDTSCAQKYNGGRGFCNSFGNYNFCQCEYPC

>LCR76

MKSSMQLISTLFFLVILVVAPGMKMVVEGQPQLCETKSLNYRGLCLKWRSCKRVCISEGFPDGRCKGFFNNKCVCRKPCALLSTEN

>LCR80

MERIPSLASLVSLLIIFATVVNQTRASICNDRLGLCDGCDQRCKAKHGPSCESKCDGPVGMLLCTCTYECGPTKLCNGGLGNCGESCNEQCCDRNCAQRYNGGHGYCNTLDDFSLCLCKYPC

>LCR66

MKVSPRLNSALLLLFMILATVMGLVTVEARTCETSSNLFNGPCLSSSNCANVCHNEGFSDGDCRGFRRRCLCTRPC

>LCR68

MKLSVRFISAALLLFMVFIATGMGPVTVEARTCESKSHRFKGPCVSTHNCANVCHNEGFGGGKCRGFRRRCYCTRHC

>LCR70

MKFSMRLISAVLFLVMIFVATGMGPVTVEARTCASQSQRFKGKCVSDTNCENVCHNEGFPGGDCRGFRRRCFCTRNC

>LCR72

MKLSLRLISALLMSVMLLFATGMGPVEARTCESPSNKFQGVCLNSQSCAKACPSEGFSGGRCSSLRCYCSKAC

>LCR75

MKSSTTSMQLIPTLFFLTILLASPEMVEGQQMCEAKSLDWKGMCLKWRNCRQVCISEGFTDGRCKGFTRKCICSKPCFVLPN

>LCR74

MENKFFAAFFLLLVLFSSQEIIGGEGRTCQSKSHHFKYMCTSNHNCAIVCRNEGFSGGRCHGFHRRCYCTRLC

>PDF1.5

MAKFCTTITLILVALVLFADFEAPTIVKAELCKRESETWSGRCVNDYQCRDHCINNDRGNDGYCAGGYPWYRSCFCFFSC

>DmAMP1

ELCEKASKTWSGNCGNTGHCDNQCKSWEGAAHGACHVRNGKHMCFCYFNC

>HsAFP1

DGVKLCDVPSGTWSGHCGSSSKCSQQCKDREHFAYGGACHYQFPSVKCFCKRQC

>Ct-AMP1

NLCERASLTWTGNCGNTGHCDTQCRNWESAKHGACHKRGNWKCFCYFNC

>EGAD1

MEHSRRMLPAILLLLFLLMPSEMGTKVAEARTCESQSHKFQGTCLRESNCANVCQTEGFQGGVCRGVRRRCFCTRLC

>SRP_SOYBN

MEMRKSCGFFFLLLLLVFASQVVVQTEGRVCESQSHGFHGLCNRDHNCALVCRNEGFSGGRCKRSRRCFCTRIC

>SPI1B

MADKGVGSRLSALFLLVLLVISIGMMQLEPAEGRTCKTPSGKFKGVCASRNNCKNVCQTEGFPSGSCDFHVANRKCYCSKPCP

>AX1

AICKKPSKFFKGACGRDADCEKACDQENWPGGVCVPFLRCECQRSC

>Fabatin-1

LLGRCKVKSNRFHGPCLTDTHCSTVCRGEGYKGGDCHGLRRRCMCLC

>Defensin

MPSYKKLVIVGFALTLLLVSFGMDASAKLCSTTMDLLICGGAIPGAVNQACDDTCRNKGYTGGGFCNMKIQRCVCRKPCALEEQTEARAGDEAAGGAGDMMSRTMAD

>Drosomycin

MMQIKYLFALFAVLMLVVLGANEADADCLSGRYKGPCAVWDNETCRRVCKEEGRSSGHCSPSLKCWCEGC

>VrD2

MEKKSLAGLCFLFLVLFVAQEVMVQTEAKTCENLANTYRGPCFTTGSCDDHCKNKEHLRSGRCRDDFRCWCTRNC

>AX2

ATCRKPSMYFSGACFSDTNCQKACNREDWPNGKCLVGFKCECQRPC

**Unclassified_40**

>AgCRP

MDTKKVYCVLYILLLTMTTPALCVQQQVEEKLLAQQEPNHDSQVRERRSVAVGPLALYAGVAVSPWVWVALVAAYGLSMVIRYGVRRTDSDSHNCANNRGWCRPFCFSH

>TEWP

MKLSVFVLVAVMLVLLCCAMQTEARRRCRSCVPFCGSNERMISTCFSGGVVCCPR

>BmTXKs2

MKIAFIVAISLAFLAVTSCIEFEKSTESHDIQKRGVTITVKPPFPGCVFYECIANCRSRGYKNGGYCTINGCQCLR

>BmDefensinB

DDTPSSRCGSGGWGPCLPIVDLLCIVHVTVGCSGGFGCCRIG

>BmDefA

MAHQRKSLVIFIFLTVLVFVFALPRDATVFDNQHSEVAIEKSTSKIDSSDVKIPGRIWCEFEEATETAICQEHCLPKGYSYGICVSNTCSCI

>Pelovaterin

MKGVYLIFTLILVYVASTWASLDAADEVRVMNVESQRLFRSRRALPCAKKSCDSWCRRLDYPGGECVTKWKCSCNWMQIDK

>Gallerimycin

MTYAILIIVSLLLISDRISNVVDKYCSENPLDCNEHCLKTKNQIGICHGANGNEKCSCMES

>Defensin

EKKCPGRCTLKCGKHERPTLPYNCGKYICCVPVKVK

>Bigdefensin

MKVFLIVLFVTIFVMVAANQEEPEQSLVEGVPLDGKIYRQARGICYVLTCNSLCFPKLGRCSYNTCYCY

>Hemocytedefensin

RCDGSSQPGWEQHGFCDNGQPCHERCKDAGFTCGGCGGVFWNQCWCCNNSC

>AfusinN

LCDGTPDDTWRHLNSCSYPNDPDCNTRCQAAGFGCGGCGGFLYGECWCCSNDCR

>Cglosin-1N

MRFFGGVIATFVVCSSLADAFCHDSISCMVGGDNVCNNVCVRQGNPNGGRCLPRDGCPGNDICACYPQSKRSDEVIDGDASIREVLKDFGIDGGAEKELNAREKRSISCNFPDPFGGLICENHCAYIGKPGGQCSDQKVCTCN

>AorsinN

MRFALFTPLLAALPLASALCHNSISCALGGDNVCNNVCVRQGNDNGGRCLPRDGCPGYDICACYPRSKRSDEVIDGDAPLREVLKDMGVEDVVNKVDARDVKLDKRSICCSFPDPWGGFCCEDHCSYIGKPGGQCSDKKVCTCN

>Nefisin-1N

MRFTTIVTPLLASATLASALCHNSISCMMGGDSTCNNVCVRQGNPSGGRCLPRDGCPGYDICACYPNSKRSDEVIDGDEPLREALNNMGIDEAAAVKNLDTRDATFDKRSICCSFPDPWGGLCCEDHCSYIGKPGGQCSDKSVCTCN

>Aflasin-1N

MRFILSTLAAGLLATAPLALAQDCSSVVCVLGDGACNRVCEMEGHTEGGKCVPRDGCPAGSEICVCGAKKAKRAVVAADGDELLARALDGVATVDQFVDAVFEKRDGSEAAVDKRSVCCSFPDPVGGLCCEAHCQQIGHLEGGQCTAQNVCVCG

>Aflasin-3

KCNRHCRSIGCRAGYCDFWTFYRRCT

>Rorsin-1

MFSESTMRSLTILLVCVLTFSVFTVTVALPPRIIHHNTGHAYGCPDDYSRCQQHCSATFGWTGWCGGVTRGECKCRE

>Rorsin-2

MAESTTTCFLLLVTGYVTAVMSEEAHLRSRRDFGCGQGMIFMCQRRCMRLYPGSTGFCRGFRCMCDTHIPLRPPFMVG

>Defensin-4

PFVDGPLCTPGKVTRPQGLVESIVGTTTGDDAPPNFSAAVVVRRNIFCRNFGTARGCYGTAHPLPRMLSCVPPKGGVFCPLVAANTHRLHCCSN

>Def

MKLSLPLVLLFALLGLFVTVAVGQTPCSSAKKVRCNVHCRGYTKLGSCYDDNCSCVDKPAAMKASFAA

>Longicornsin

TPHQKTCHPLKEAHANAVCKEYCGSVGYLLGECGKEGICVCEKRQLNE

>AM-135_Def

APHNKSCHRLKDPHANAVCKAYCGKAGYKLGECGLQGICICKKTKISTKVSKSSK

>AM-1246_Def

MKSSMLLLVCVTFLVIVSSPQNGVLADKLIGSCVWGAVDYTSNCNAECVRRGLR

>PJD1

MGSKVYYVLMLALSFYVVQLCAFPRNSVAFEKQHESLPHADYIVTQKTESHEKTTAEPKLPGRIWCQYEEVTEDAICQEHCIPKGYSYGLCISNTCSCI

>PJD2

MELKTVNLFLLIALSICFVQNAIAMPRDSAAIHDESVVPISSEALAAKEDTTKLEKSTTELTIPGRISCKYEEPTDDTICQEHCLPKGYNYGICVSYTCSCI

>TY1

MGVKVINVFLLIAVSACLIHAVAGKPNPRDSSVVEEQSLGPIHNEDLEVKVKPETTTTPEPRIPGRVSCDFEEANEDAVCQEHCLPKGYTYGICVSHTCSCI

>TY2

MQNMSKLVFTKSILILLFVVITIILGSSEVHELQGESCSGAYPYTDCQEKCKSSSYSDNNAYCAMKNGQYRCYCYALSDDDVVWDN

>Defensin

MMKIAVPVFLILALFAMSQAAPSLALPGTVQVANVQPRGYCNLSNCLSQCYRRGFRWGWCDFFNTCHCS

>Spodoptericin

MNFLKVFLVICAILATVWASPLGASEDREHHPEERAPCAQSEKDRCVAFCLTNGFSGGFCTSRGCKCEE

>Defensin

MKTKAIVMLMLLVLVAATLVQGEPEPSYILDCRTNGGRCVTGYCSNTLPYSCGGGAICCRHAYG

>DEF1

MKTKAIVMLMLLVLVAATLVQGEPEPSYFNDCGSNGGSCTRGYCSYSNRLPYTCSLGRTCCRLAYV

>DEF2

MRCLTVTFVCLLVLGAFALSTVDGRRHPTGHDYGCPVFQIECQQHCSATFGWQGRCGGSRRSECICRN

>Def

MKFLQIFVLIATLWIAIVTANPVDDQKTAEIQENSNLIDETNNDEVIIQPRLSCQALGPIGCAANCKRLGFRGGWCTTGNTCRCFR

>Def

MNTKLIIFLATLVILTDAYVVKSPKDLSSADSNALEKNSQSLLHATYDESLYIPMRVSSCSDGICDLGCKILGYPHGRCISANTCQCY

>DLP-2

IMFFEMQACWSHSGVCRDKSERNCKPMAWTYCENRNQKCCEY

>DLP-1

FVQHRPRDCESINGVCRHKDTVNCREIFLADCYNDGQKCCRK

>DEFL3

MRLLFLLFLLLVCLIQMASGHEKTGRKHECQNMGGACKHQKTHGCAILPADCKSRNKHCCRV

>CBD138

MRLLFLLFLLLVCLVQMTSGREKRRKSLECERMGGVCKHQKTHGCSILPAECKSRNKHCCRV

>Bigdefensin

MKGNIGIAVFYMLLLLLPTDSIGKKMEEEQEKLFRQKRNPLIPAIYIGATVGPSVWAYLVALVGAAAVTAANIRRASSDNHSCAGNRGWCRSKCFRHEYVDTYYSAVCGRYFCCRSR

>AiBD

MTRPSLVRCYSLFFTALIVMAIICPAWSEEIPKSRKKRAIPIAYVGMAVAPQVFRWLVRAYGAAAVTAAGVTLRRVINRSRSNDNHSCYGNRGWCRSSCRSYEREYRGGNLGVCGSYKCCVT

**Vertebrate_157**

>DEFA4

MRIIALLAAILLVALQVRAGPLQARGDEAPGQEQRGPEDQDISISFAWDKSSALQVSGSTRGMVCSCRLVFCRRTELRVGNCLIGGVSFTYCCTRVD

>DEFA5

MRTIAILAAILLVALQAQAESLQERADEATTQKQSGEDNQDLAISFAGNGLSALRTSGSQARATCYCRTGRCATRESLSGVCEISGRLYRLCCR

>DEFA6

MRTLTILTAVLLVALQAKAEPLQAEDDPLQAKAYEADAQEQRGANDQDFAVSFAEDASSSLRALGSTRAFTCHCRRSCYSTEYSYGTCTVMGINHRFCCL

>MNP1A

MRTLAILAAILLVALQAQAEPLQARTDEATAAQEQIPTDNPEVVVSLAWDESLAPKDSVPGLRKNMACYCRIPACLAGERRYGTCFYLGRVWAFCC

>MNP2

MRTLAILAAILLFALLAQAKSLQETADDAATQEQPGEDDQDLAVSFEENGLSTLRASGSQARRTCRCRFGRCFRRESYSGSCNINGRIFSLCCR

>DEFA4

MRTLTILAAILLFTLQAQAESLQERADEVATQEQPGKDDQDLAVSFEENGLSTLRASGSQARRTCYCRTGRCYTPEFHSGKCVFNGRTYKLCCR

>Defcr-rs1

MKTLVLLSALVLPCFQVQADPIQNTDEETKTEEQPEEEDQAVSVSFGGTEGSALQDVAQRRFPWCRKCRVCQKCQVCQKCPVCPTCPQCPKQPLCEERQNKTAITTQAPNTQHKGC

>Defcr-rs12

MKKLVLLSAFVLLAFQVQADSIQNTDEEIKTEEQPGEENQAVSISFGDPEGYALQDAAIRRARRCPPCPSCLSCPWCPRCLRCPMCKCNPK

>Defcr-rs7

MKKLVLLFALVLLAFQVQADSIQNTDEETKTEEQQGEEDQAVSVSFGDPQGSGLQDAALGWGRRCPRCPPCPRCSWCPRCPTCPRCNCNPK

>Cryptdin9

MKTLVLLSALVLLAFQVQADPIQNTDEETKTEEQPGEEDQAVSVSFGDPEGSSLQEESLRDLVCYCRKRGCKRREHMNGTCRKGHLLYMLCCR

>DEFA7

MRTLTLLSAFLLVALQAWAEPLQARADEMPAQKQPPADDQDVVIYFSGDDSSSLQVPGSTKGLICHCRVLYCLFGEHLGGTSFIHGERYPICCY

>Entericdefensinalpha5

MKTLVLLSALVLLALQVQAEPTPKTDEGTKTDEQPGKEDQVVSVSIEGQGDPAFQDAVLRDLKCFCRAKSCNWGEGIMGICNKRYGSLILCCR

>Cryptdin5

MKTFVLLSALVLLAFQVQADPIHKTDEETNTEEQPGEEDQAVSISFGGQEGSALHEELSKKLICYCRIRGCKRRERVFGTCRNLFLTFVFCCS

>RatNP-4

MRTLTLLITLLLLALHTQAESPQERAKAAPDQDMVMEDQDIFISFGGYKGTVLQDAVVKAGQACYCRIGACVSGERLTGACGLNGRIYRLCCR

>Cryptdin4

MKTLVLLSALVLLAFQVQADPIQNTDEETKTEEQPGEEDQAVSISFGGQEGSALHEKSLRGLLCYCRKGHCKRGERVRGTCGIRFLYCCPRR

>HANP-4

VTCFCKRPVCDSGETQIGYCRLGNTFYRLCCRQ

>HANP-3

VTCFCRRRGCASRERLIGYCRFGNTIYGLCCRR

>RatNP-2

MRTLTLLTALLLLALHTQAKSPQGTAEEAPDQEQLVMEDQDISISFGGDKGTALQDADVKAGVTCYCRSTRCGFRERLSGACGYRGRIYRLCCR

>RK-1

MPCSCKKYCDPWEVIDGSCGLFNSKYICCREK

>NP-1

MRTLALLAAILLVALQAQAEHVSVSIDEVVDQQPPQAEDQDVAIYVKEHESSALEALGVKAGVVCACRRALCLPRERRAGFCRIRGRIHPLCCRR

>NP-3b

GRCVCRKQLLCSYRERRIGDCKIRGVRFPFCCPR

>NP-4

MRTLALLAAILLVTLQAQAELHSGMADDGVDQQQPRAQDLDVAVYIKQDETSPLEVLGAKAGVSCTCRRFSCGFGERASGSCTVNGVRHTLCCRR

>NP-3a

MRTLILLAAILLAALQAQAELFSVNVDEVLDQQQPGSDQDLVIHLTGEESSALQVPDTKGICACRRRFCPNSERFSGYCRVNGARYVRCCSRR

>GNCP-1A

MRTVPLFAACLLLTLMAQAEPLPRAADHSDTKMKGDREDHVAVISFWEEESTSLEDAGAGAGRRCICTTRTCRFPYRRLGTCIFQNRVYTFCC

>Defa11

MGTLTLLTTLLLLALHTQAESPQGCTEDQKLLIMEDQDISISFTGDKGTALQDADVKSGVICNCRPRGCSFKERVYGECGYNGHILRLCCQPLNTMNKKSPSVI

>Defa7

MRTLTLLTTLLLLALHTQAESPQGSRSTEEALDQEQLVMEDQDISISFGGDKGTALQDADVKSGVTCYCRLLSCQFGERLAGSCRSGGVTYPLCCH

>Defa6

MKTLVLLSALVLVAYQVQADPIQGAEEETKTEEQPSDEDQDVSVSFEGPEASALQDFEIGRPVRRCRCRANCGPKEYATAFCAQGPFKQFKFCCT

>Defa10

MRTLTLLTALLLLALHTQAESPQGSPKEAPDQEQLDMEDQDISVFFGGDKGTALQDAAGSTCSCRIGTCVSGEWLSWVCRINGRIYRLCCR

>Defa9

MKTLVLLSALVLLAFQIQADPIQEAEEETKTEEQPADEDQDVSVSFEGPEPSALQNLEIRWPWKRCHCRSFCRPYENATSFCAQGLFKQHKFCCLETWPPRMK

>Defa8

MKTLVLLSALVLLAFQVQADHIQEAEEETKTEEQPADEDQDMSVSFEGPEASALQNLEIGWPLKQCHCRKFCRPYEKAEGSCRPGLFIKRKICCIQQWTPGRT

>Defa-rs1

MKTLILLSALVLLALQVQADPIQEAEEETKTEEQPADEDQDVSVSFEGPEASAVQDLRVRRTLQCSCRRVCRNTCSCIRLSRSTYAS

>DEFB1

MRTSYLLLFTLCLLLSEMASGGNFLTGLGHRSDHYNCVSSGGQCLYSACPIFTKIQGTCYRGKAKCCK

>DEFB2

MRVLYLLFSFLFIFLMPLPGVFGGIGDPVTCLKSGAICHPVFCPRRYKQIGTCGLPGTKCCKKP

>DEFB103A

MRIHYLLFALLFLFLVPVPGHGGIINTLQKYYCRVRGGRCAVLSCLPKEEQIGKCSTRGRKCCRRKK

>DEFB104A

MQRLVLLLAVSLLLYQDLPVRSEFELDRICGYGTARCRKKCRSQEYRIGRCPNTYACCLRKWDESLLNRTKP

>DEFB105A

MALIRKTFYFLFAMFFILVQLPSGCQAGLDFSQPFPSGEFAVCESCKLGRGKCRKECLENEKPDGNCRLNFLCCRQRI

>DEFB106A

MRTFLFLFAVLFFLTPAKNAFFDEKCNKLKGTCKNNCGKNEELIALCQKSLKCCRTIQPCGSIID

>DEFB108B

MRIAVLLFAIFFFMSQVLPARGKFKEICERPNGSCRDFCLETEIHVGRCLNSQPCCLPLGHQPRIESTTPKKD

>DEFB110

MKIQLFFFILHFWVTILPARSNFEPKYRFERCEKVRGICKTFCDDVEYDYGYCIKWRSQCCV

>DEFB111

MKIQLFFFILHFWVTILPAKKKYPEYGSLDLRRECRIGNGQCKNQCHENEIRIAYCIRPGTHCCLQQ

>DEFB112

MKLLTTICRLKLEKMYSKTNTSSTIFEKARHGTEKISTARSEGHHITFSRWKSCTAIGGRCKNQCDDSEFRISYCARPTTHCCVTECDPTDPNNWIPKDSVGTQEWYPKDSRH

>DEFB113

MKILCIFLTFVFTVSCGPSVPQKKTREVAERKRECQLVRGACKPECNSWEYVYYYCNVNPCCAVWEYQKPIINKITSKLHQK

>DEFB114

MRIFYYLHFLCYVTFILPATCTLVNADRCTKRYGRCKRDCLESEKQIDICSLPRKICCTEKLYEEDDMF

>DEFB115

MLPDHFSPLSGDIKLSVLALVVLVVLAQTAPDGWIRRCYYGTGRCRKSCKEIERKKEKCGEKHICCVPKEKDKLSHIHDQKETSELYI

>DEFB116

MSVMKPCLMTIAILMILAQKTPGGLFRSHNGKSREPWNPCELYQGMCRNACREYEIQYLTCPNDQKCCLKLSVKITSSKNVKEDYDSNSNLSVTNSSSYSHI

>DEFB117

CRSQKSCWIIKGHCRKNCKPGEQVKKPCKNGDYCCIPSNTDS

>DEFB118

MKLLLLALPMLVLLPQVIPAYSGEKKCWNRSGHCRKQCKDGEAVKDTCKNLRACCIPSNEDHRRVPATSPTPLSDSTPGIIDDILTVRFTTDYFEVSSKKDMVEESEAGRGTETSLPNVHHSS

>DEFB119

MKLLYLFLAILLAIEEPVISGKRHILRCMGNSGICRASCKKNEQPYLYCRNCQSCCLQSYMRISISGKEENTDWSYEKQWPRLP

>DEFB121

MKLLLLLLTVTLLLAQVTPVMKCWGKSGRCRTTCKESEVYYILCKTEAKCCVDPKYVPVKPKLTDTNTSLESTSAV

>DEFB122

MKPFLVTLAVLLLFFQVTAVGSIEKCWNFRGSCRDECLKNEKVYVFCMSGKLCCLKPKDQPHLPQRTKN

>DEFB123

MKLLLLTLTVLLLLSQLTPGGTQRCWNLYGKCRYRCSKKERVYVYCINNKMCCVKPKYQPKERWWPF

>DEFB124

EFKRCWKGQGACQTYCTRQETYMHLCPDASLCCLSYALKPPPV

>DEFB125

MLTFIICGLLTRVTKGSFEPQKCWKNNVGHCRRRCLDTERYILLCRNKLSCCISIISHEYTRRPAFPVIHLEDITLDYSDVDSFTGSPVSMLNDLITFDTTKFGETMTPETNTPETTMPPSEATTPETTMPPSETATSETMPPPSQTALTHN

>DEFB126

MKSLLFTLAVFMLLAQLVSGNWYVKKCLNDVGICKKKCKPEEMHVKNGWAMCGKQRDCCVPADRRANYPVFCVQTKTTRISTVTATTATTTLMMTTASMSSMAPTPVSPTG

>DEFB127

MGLFMIIAILLFQKPTVTEQLKKCWNNYVQGHCRKICRVNEVPEALCENGRYCCLNIKELEACKKITKPPRPKPATLALTLQDYVTIIENFPSLKTQST

>DEFB128

MKLFLVLIILLFEVLTDGARLKKCFNKVTGYCRKKCKVGERYEIGCLSGKLCCANDEEEKKHVSFKKPHQHSGEKLSVLQDYIILPTITIFTV

>DEFB129

MKLLFPIFASLMLQYQVNTEFIGLRRCLMGLGRCRDHCNVDEKEIQKCKMKKCCVGPKVVKLIKNYLQYGTPNVLNEDVQEMLKPAKNSSAVIQRKHILSVLPQIKSTSFFANTNFVIIPNATPMNSATISTMTPGQITYTATSTKSNTKESRDSATASPPPAPPPPNILPTPSLELEEAEEQ

>DEFB130

MKLHSLISVLLLFVTLIPKGKTGVIPGQKQCIALKGVCRDKLCSTLDDTIGICNEGKKCCRRWWILEPYPTPVPKGKSP

>DEFB131

MRVLFFVFGVLSLMFTVPPGRSFISNDECPSEYYHCRLKCNADEHAIRYCADFSICCKLKIIEIDGQKKW

>DEFB32

MKFLLLVLAALGFLTQVIPASAGGSKCVSNTPGYCRTCCHWGETALFMCNASRKCCISYSFLPKPDLPQLIGNHWQSRRRNTQRKDKKQQTTVTS

>DEFB135

MKPLLVVFVFLFLWDPVLAGINSLSSEMHKKCYKNGICRLECYESEMLVAYCMFQLECCVKGNPAP

>DEFB136

MATRSVLLALVVLNLLFYVPPGRSGPNVYIQKIFASCWRLQGTCRPKCLKNEQYRILCDTIHLCCV

>DEFB137

MNLCLSALLFFLVILLPSGKGMFGNDGVKVRTCTSQKAVCFFGCPPGYRWIAFCHNILSCCKNMTRFQPPQAK

>DEFB1

DFASCHTNGGICLPNRCPGHMIQIGICFRPRVKCCRSW

>DEFB3

LALLFLVLSAGSGFTQGVRNHVTCRINRGFCVPIRCPGRTRQIGTCFGPRIKCCRSW

>DEFB5

MRLHHLLLVLLFLVLSAGSGFTQVVRNPQSCRWNMGVCIPISCPGNMRQIGTCFGPRVPCCRRW

>DEFB10

MRLHHLLLLLLLVVLSSGSGFTQGVRSYLSCWGNRGICLLNRCPGRMRQIGTCLAPRVKCCR

>DEFB11

MRLHHLLLALLFLVLSAGSGISGPLSCRRNGGVCIPIRCPGPMRQIGTCFGRPVKCCRSW

>EBD

MRLHHLLLTLLFLVLSAGSGFTQGISNPLSCRLNRGICVPIRCPGNLRQIGTCFTPSVKCCRWR

>DEFB1

MRLHHLLLVLFFLVLSAGSGFTQGIRSRRSCHRNKGVCALTRCPRNMRQIGTCFGPPVKCCRKK

>DEFB1

MRIHYLLFAVLFLFLMPVPGEGGIINTIQRYFCRVRGGRCAALTCLPRETQIGRCSVKGRKCCRTRK

>Defb1

MKTHYFLLVMICFLFSQMEPGVGILTSLGRRTDQYKCLQHGGFCLRSSCPSNTKLQGTCKPDKPNCCKS

>DEFB1

MRLHRLLLVFLLMVLLPVPGLLKNIGNSVSCLRNKGVCMPGKCAPKMKQIGTCGMPQVKCCKRK

>GAL1

MRIVYLLLPFILLLAQGAAGSSQALGRKSDCFRKSGFCAFLKCPSLTLISGKCSRFYLCCKRIWG

>Osp-1

LFCRKGTCHFGGCPAHLVKVGSCFGFRACCKWPWDV

>Defb2

MRTLCSLLLICCLLFSYTTPAVGSLKSIGYEAELDHCHTNGGYCVRAICPPSARRPGSCFPEKNPCCKYMK

>Defb4

MRIHYLLFSFLLVLLSPLSAFTQSINNPITCLTKGGVCWGPCTGGFRQIGTCGLPRVRCCKKK

>DEFB2

MRLHHLLLVLFFVVLSAGSGFTHGVTDSLSCRWKKGICVLTRCPGTMRQIGTCFGPPVKCCRLK

>Defb3

MRIHYLLFAFLLVLLSPPAAFSKKINNPVSCLRKGGRCWNRCIGNTRQIGSCGVPFLKCCKRK

>Defb4

MRIHYLLFTFLLVLLSPLAAFTQIINNPITCMTNGAICWGPCPTAFRQIGNCGHFKVRCCKIR

>Defb5

MRIHYLLFAFLLVLLCPLASDFSKTINNPVSCCMIGGICRYLCKGNILQNGNCGVTSLNCCKRK

>Defb6

MKIHYLLFAFILVMLSPLAAFSQLINSPVTCMSYGGSCQRSCNGGFRLGGHCGHPKIRCCRRK

>Defb7

MRIHYVLFAFLLVLLSPFAAFSQDINSKRACYREGGECLQRCIGLFHKIGTCNFRFKCCKFQIPEKKTKIL

>Defb8

MRIHYLLFTFLLVLLSPLAAFSQKINEPVSCIRNGGICQYRCIGLRHKIGTCGSPFKCCK

>Defb9

MRTLCSLLLICCLLFSYTTPAANSIIGVSEMERCHKKGGYCYFYCFSSHKKIGSCFPEWPRCCKNIK

>Defb10

MRTLCSLLLICCLLFSYTTPAVGDLKHLILKAQLTRCYKFGGFCHYNICPGNSRFMSNCHPENLRCCKNIKQF

>Defb11

MRTLCSLLLICCLLFSYTTPAVGDLKHLILKAQLARCYKFGGFCYNSMCPPHTKFIGNCHPDHLHCCINMKELEGST

>Defb12

MKNLPSNMALSREVFYFGFALFFIVVELPSGSWAGLEYSQSFPGGEIAVCETCRLGRGKCRRTCIESEKIAGWCKLNFFCCRERI

>Defb13

MRIFSLIVAGLVLLIQLYPAWGTLYRRFLCKKMNGQCEAECFTFEQKIGTCQANFLCCRKRKEH

>Defb14

MRLHYLLFVFLILFLVPAPGDAFLPKTLRKFFCRIRGGRCAVLNCLGKEEQIGRCSNSGRKCCRKKK

>Defb19

MRLALLLLAILVATELVVSGKNPILQCMGNRGFCRSSCKKSEQAYFYCRTFQMCCLQSYVRISLTGVDDNTNWSYEKHWPRIP

>Defb29

MPVTKSYFMTVVVVLILVDETTGGLFGFRSSKRQEPWIACELYQGLCRNACQKYEIQYLSCPKTRKCCLKYPRKITSF

>Defb34

MKTFLFLFAVLFFWSQPRMHFFFFDEKCSRINGRCTASCLKNEELVALCWKNLKCCVTVQSCGRSKGNQSDEGSGHMGTRG

>Defb35

MPQTFFVFCFLFFVFLQLFPGTGEIAVCETCRLGRGKCRRACIESEKIVGWCKLNFFCCRERI

>Defb37

MKFSYFLLLLLSLSNFQNNPVAMLDTIACIENKDTCRLKNCPRLHNVVGTCYEGKGKCCHKN

>Defb38

MKISCFLLLILSLYFFQINQAIGPDTKKCVQRKNACHYFECPWLYYSVGTCYKGKGKCCQKRY

>Defb39

MKISYFLLLILSLGSSQINPVSGDDSIQCFQKNNTCHTNQCPYFQDEIGTCYDKRGKCCQKRLLHIRVPRKKKV

>Defb40

MKISCFLLMIFFLSCFQINPVAVLDTIKCLQGNNNCHIQKCPWFLLQVSTCYKGKGRCCQKRRWFARSHVYHV

>Defb41

MKFHLFFFILLFGATILTARSHIDIKNGIERCEKVRGMCKTVCDIDEYDYGYCIRWRNQCCI

>Defb50

MKTLCFLLLTSGLLYLMVKGVGSHPGTFHVRIKCMPKMTAVFGDNCSFYSSMGDLCNNTKSVCCMVPVRMDNI

>DEFB107A

MKIFFFIFAALILLAQIFQARTAIHRALISKRMEGHCEAECLTFEVKTGGCRAELAPFCCKNRKKH

>DEFB120

MKFLFLFLAILLATEVPVISVECWMDGHCRLLCKDGEDSIIRCRNRKRCCVPSRYLTIQPVTIHGILGWTTPRMSTTAPQPKRNIHNG

>DEFB126

MKSLLFTLAVFMLLAQLVSGNLYVKRCLNDIGICKKTCKPEEVRSEHGWVMCGKRKACCVPADKRSAYPSFCVHSKTTKTSTVTARATATTATTATAATPLMISNGLISLMTTMAATPVSPTT

>Sphe-2

SFGLCRLRRGFCARGRCRFPSIPIGRCSRFVQCCRRVW

>Osp-2

APGNKAECEREKGYCGFLKCSFPFVVSGKCSRFFFCCKNIW

>Osp-3

IPRPLDPCIAQNGRCFTGICRYPYFWIGTCRNGKSCCRRR

>Osp-4

LPVNEAQCRQVGGYCGLRICNFPSRFLGLCTRNHPCCSRVWV

>TAP

MRLHHLLLALLFLVLSASSGFTQGVGNPVSCVRNKGICVPIRCPGNMKQIGTCVGRAVKCCRKK

>GAL8

MKILYFLLAVLLTVLQSSLGFMRVPNNEAQCEQAGGICSKDHCFHLHTRAFGHCQRGVPCCRTVYD

>GAL9

MRILFFLVAVLFFLFQAAPAYSQEDADTLACRQSHGSCSFVACRAPSVDIGTCRGGKLKCCKWAPSS

>GAL3

MKILYLLIPFFLLFLQGAAGTATQCRIRGGFCRVGSCRFPHIAIGKCATFISCCGRAYEVDALNSVRTSPWLLAPGNNPH

>GAL2

MRILYLLFSLLFLALQASPGLSSPRRDMLFCKGGSCHFGGCPSHLIKVGSCFGFRSCCKWPWNA

>GAL12

MRNLCFVFIFISLLAHGSTHGPDSCNHDRGLSRVGNCNPGEYLAKYCFEPVILCCKPLSPTPTKT

>Defb1

MRILHFLLAFLIVFLLPVPGFTAGIETSFSCSQNGGFCISPKCLPGSKQIGTCILPGSKCCRKK

>CBD1

MRPLYLLLLLLCLLFSYLPPGAGFLTGIGQRSDQYICARKGGTCNFSPCPLFTRIDGTCYRGKAKCCMP

>THP1

MRIVYLLFPFILLLAQGAAGSSLALGKREKCLRRNGFCAFLKCPTLSVISGTCSRFQVCCKTLLG

>Defb38

MKISCFLLLVLSLYLFQVNQATDQDTAKCVQKKNVCYYFECPWLSISVSTCYKGKAKCCQKRY

>Defb37

MKISCFLLLVLSLSCFQINPFAVLDTRVCIEKRNTCHILQCPLFRDVVGTCFEGIGKCCHKYF

>Defb17

MKFHLLFFILLFSITILTGKRSYPEYGSLDLRKECRMSKGHCKLQCSENEIRIAFCIRPGTHCCI

>Defb42

MRLYLLLSTLLFLLGLLPRVRSGLGAAETHCVNLQGICRRDICKLIEDEIGACRRRWKCCRLWWVLLPIPTPVIFSDYQEPLQTKMK

>Defb30

MGSLQLILVLFVLLSDVPPVRSGVNMYIRQIYDTCWKLKGHCRNVCGKKEIFHIFCGTQFLCCIERKEMPVLFVK

>Defb18

MQSAMKLFFIFLIFVFSVSCGPSAPQMKTRDVLERTHKCFLVGGECKSECSSWEYEYVFCYTGPCCVMREYKRVEKFSNTPKYTT

>Defb49

MKLPVLFLLFCFLDLLKTVKAEMKDTLFCFLKKGKCRHVCMNVEKRVGPCTKLNANCCIFVRDMRAIIPEDQRTVSIKIRNKQN

>Defb44

MDLHLLCLLLFLVTSLPEGYCVIGNSGVSFKPCTSEGGYCFFGCKLGWIWITYCNNIMSCCKKDTKHSLPQTKGV

>Defb43

MRLLLSILGVLTLLSILPLARSFLANQECFSEYRHCRMKCKANEYAIRYCADWTICCRVKKREAKKKIMW

>Defb41

MRSHSFLSALFLLVMMMPRGKAGVVPGEKQCILLKGVCKDVSCTSTDDTIGVCNDEKKCCRRWWVFDPYPTPVPKGKSP

>Defb33

MRLLFLLFLLLVCLAQKTSGRKRNTKFRQCEKMGGICKYQKTHGCSILPAECKSRYKHCCRL

>Defb39

MKISCFLLLVLSLSCFQINSVSGIDSVKCFQKNNTCHTIRCPYFQDEVGTCYEGRGKCCQKRLLSIRVPKKKV

>Defb15

MKTFLFLFAVFFFLDPAKNAFFDEKCSRINGRCTESCLKNEELIALCQKNLKCCVTVQPCGRDKGDELDEDSGYNRTRG

>Defb25

MAKWILLIVALLVLGHVPSGSTEFKRCWNGQGACRTYCTRQEKFIHLCPDASLCCLSYSLKASPHSRAGGV

>Defb27

MKTAVLTMVLLLLLSQVIPGSPEKCWKSFGICREECLRKEKFYIFCWDGSLCCVKPKNVPQWSQSSE

>Defb20

MKLPQLLLILLFVVLADSVQPKRCFSNVAGYCRKRCRLVEISEMGCLHGKYCCVNELENKRHKKDTVVEQPMEPRDKSKVQDYMVLPTITYYTITI

>Defb36

MKLLLLTLAALLLLSQLTPGDAQKCWNLHGKCRHRCSRKESVYVYCTNGKMCCVKPKYQPKPKPWMF

>Defb50

MKTLHLLLLISGLLSVFVKGVGSHPGTVHVRFKCIPKIAAVFGDNCPFYGNVDGLCNDKKSVCCMVPVRLDNI

>Defb40

MKISCFLMLVLFLSCFQMNSGAGLDTMKCVRGKNNCHMHRCPWFFVLISTCYSGKGSCCQKRRWFTRSHVNNV

>Defb21

MRLLLMALPLLALLPQVIPDYSAEKRCLNRLGHCKRKCKAGEMVMETCKYFQVCCVLDDNDYKQKASITRTMEKTSTIEYNLS

>Defb10

MKTLCSLLLIGCLLFSYDTPVVGELKHLGMTAETEWCRLFEGFCHDKNCPPPTSHVGSCHPEKRSCCKDRR

>Defb19

CRKSCWVIRGHCRKHCRSGERVKKPCSNGDYCCIAKKINTVPQAPKNAFSRNFRVHSRTAPVAVLKNST

>Defb9

MRTLCSLLLICCLLFSYDTPVVGELKHLGLKTEFEQCQRIRGYCLNTYCRFPTSHVGSCYPEKRYCCKNIR

>Defb11

MRTLCSLLLIGCLLFSYDTPVVGFLRRSVSGFQECHSKGGYCYRYYCPRPHRRLGSCYPYAANCCRRRR

>Defb26

MLFPTLAFIICGLLTQVTKAAWRRMPPKCWKDNLGRCRVRCRGDERYIYLCRNKANCCILLTLAEDQSAHPKPTSVHPENVTWKNTSISPTTRYIDDITLDKTSIGTPTNVAGIGKALNLTVITPTHP

>Defb28

MLLTRSSTLSGHIKLWFLTLAVLVVLAQTSPEGWFRTCFYGMGKCRHVCRANEKKKERCGENSFCCLGETKSKLSNIPTNKGRKKD

>Defb13

MRIFSLIVAGLVLLIQLHPAKGTLYRRFLCKKMKGRCETACLSFEKKIGTCRADLTPLCCKEKKKH

>Defb23

MKLLFPLFASLVLQYQVESEFMVVKKCLMGFGKCKDSCLPEETQVQNCKSKKCCMGPKVTELIKSYLRHEIPHIPDDDIVEMMKMSENLTEEMQRQQALVAFSQSQVAKSLLSNINSAIIPNAFPVTKRTRRHYMGNTASTERHTKQSRDSANAAPLQPRPGPP

>Defb51

MRIHAFLALLAIFQVLHAITSALNFQRPCYLRGGICLKQGTPNCEPFRGPCRAFTVCCKIRS

>Defb29

MPVTKPYFVTVAVLLILVDKTTGGLFGLRSGKRREPWVSCELYQGSCRNACQKYEIQYLTCPKKRKCCLKFPMKITRV

>Spag11

MKVLLLFAVFFCLVQRNSGDIPPGIRNTVCFMQRGHCRLFMCRSGERKGDICSDPWNRCCVSSSIKNR

>Defb3

MRIHYLLFSFLLVLLSPLSAFSKKVYNAVSCMTNGGICWLKCSGTFREIGSCGTRQLKCCKKK

>Defb2

MRTLCSLLLIGCLLFSYATPVAGILGPLRIQTDYHRCLREKGFCLNAVCPRSTLFVGTCFPYKFYCCKFKR

>Defb22

MKSLLSALMIIMFLAHLVTGNWYVRKCANKLGTCRKTCRKGEYQTDPATGKCSIGKLCCILDLKLAGQCGGADGNQAAAGTQAAGGTRAAGGTQGTGGTGATGAAATTAAP

>Defb52

MKLLYLLISVVLLISQVMAAPEGCKQEGQTEWKDFVRTKGAFIHFFNNDYGHHYCDSPTSVCLRRRTNCTRMPGLCPGRSFCCVRT

>TBD-1

YDLSKNCRLRGGICYIGKCPRRFFRSGSCSRGNVCCLRFG

>Apl_AvBD2

MRILYLLFSVLFLVLQVSPGLSLPQRDMFLCRIGSCHFGRCPIHLVRVGSCFGFRSCCKSPWDV

>P15

IRNSLTCRFNFGICLPKRCPGRMRQIGTCF

>Crotasin

MKILYLLSAFLFLAFLSESGNAQPQCRWLDGFCHSSPCPSGTTSIGQQDCLWYESCCIPRYEK

>DEFT1P

MPCFSWWPCRLRRSHFRQELMKLQPRSSLEQMIRKWLMPLHGMKVPLFRFQTQREA

>Demidefensin-3

MRTLALHTAMLLLVALHAQAEARQARADEAAAQQQPGADDQGMAHSFTWPENAALPLSESERGLRCICVLGICRLL
