# Supplementary material for: iDPF-PseRAAAC: A Web-Server for Identifying the Defensin Peptide Family and Subfamily Using Pseudo Reduced Amino Acid Alphabet Composition
Source: PLoS One. 2015 Dec 29;10(12):e0145541. doi: 10.1371/journal.pone.0145541 (PMC4694767; doi:10.1371/journal.pone.0145541)
Supplement: S1 Table — (DOCX) [file pone.0145541.s002.docx]

**S1 Table. The prediction results of 10-fold cross-validation for the benchmark dataset**

--------------------------------------------------------------------------------------------------------------------

Class | TP | TN | FP | FN | ACC | MCC | Sn | Sp

Insect | 53 | 266 | 7 | 7 | 95.796 | 0.858 | 88.33 | 97.44

Invertebrate | 21 | 289 | 10 | 13 | 93.093 | 0.609 | 61.76 | 96.66

Plant | 36 | 289 | 2 | 6 | 97.598 | 0.888 | 85.71 | 99.31

Unclassified | 15 | 281 | 12 | 25 | 88.889 | 0.398 | 37.50 | 95.90

Vertebrate | 154 | 153 | 23 | 3 | 92.192 | 0.851 | 98.09 | 86.93

------------------------------------------------------------------------------------------------------------------

Overall accuracy:83.78
