# Supplementary material for: iDPF-PseRAAAC: A Web-Server for Identifying the Defensin Peptide Family and Subfamily Using Pseudo Reduced Amino Acid Alphabet Composition
Source: PLoS One. 2015 Dec 29;10(12):e0145541. doi: 10.1371/journal.pone.0145541 (PMC4694767; doi:10.1371/journal.pone.0145541)
Supplement: S2 Table — (DOCX) [file pone.0145541.s003.docx]

**S2 Table.** Results obtained by **iDPF-PseRAAAC** in identifying defensin peptide families with **(A)** single amino acid case $(\text{N}=1)$and **(B)** tripeptide case $(\text{N}=3)$

**(A): For the single amino acid case** $(\text{N}=1)$

| Family | Subset | Metrics | *N*-Peptide compositions of RAAA with S size (**N**, **S**) | | | | | |
| --- | --- | --- | --- | --- | --- | --- | --- | --- |
|  |  |  | **(1, 20)** | **(1, 13)** | **(1, 11)** | **(1, 9)** | **(1, 8)** | **(1, 5)** |
|  |  |  | 20 | 13 | 11 | 9 | 8 | 5 |
| Insect |  | Sn(%) | 71.67 | 73.33 | 73.33 | 61.67 | 61.67 | 31.67 |
|  |  | Sp(%) | 94.87 | 94.14 | 91.58 | 91.21 | 89.38 | 89.74 |
|  |  | MCC | 0.68 | 0.68 | 0.62 | 0.53 | 0.49 | 0.24 |
| Invertebrate |  | Sn(%) | 50.00 | 35.29 | 47.06 | 50.00 | 44.12 | 44.12 |
|  |  | Sp(%) | 95.65 | 95.32 | 95.65 | 95.32 | 94.98 | 92.64 |
|  |  | MCC | 0.48 | 0.35 | 0.46 | 0.47 | 0.41 | 0.35 |
| Plant |  | Sn(%) | 64.29 | 76.19 | 61.90 | 35.71 | 40.48 | 21.43 |
|  |  | Sp(%) | 98.63 | 95.19 | 94.85 | 96.91 | 96.22 | 96.91 |
|  |  | MCC | 0.72 | 0.69 | 0.57 | 0.42 | 0.44 | 0.27 |
| Unclassified |  | Sn(%) | 37.50 | 27.50 | 22.50 | 7.50 | 10.00 | 0.00 |
|  |  | Sp(%) | 96.93 | 96.59 | 94.20 | 98.29 | 96.93 | 99.32 |
|  |  | MCC | 0.43 | 0.32 | 0.20 | 0.12 | 0.12 | -0.03 |
| Vertebrate |  | Sn(%) | 96.18 | 93.63 | 87.90 | 89.17 | 85.99 | 89.17 |
|  |  | Sp(%) | 77.27 | 81.25 | 81.82 | 60.80 | 65.34 | 49.43 |
|  |  | MCC | 0.74 | 0.75 | 0.70 | 0.52 | 0.52 | 0.42 |
|  | OA(%) | | 75.98 | 73.87 | 69.97 | 63.66 | 62.46 | 54.95 |

The bold values show the best results

**(B) :For the tripeptide case** $(\text{N}=3)$

| Family | Subset | Metrics | *N*-Peptide compositions of RAAA with S size (**N**, **S**) | | | | | |
| --- | --- | --- | --- | --- | --- | --- | --- | --- |
|  |  |  | **(3, 20)** | **(3, 13)** | **(3, 11)** | **(3, 9)** | **(3, 8)** | **(3, 5)** |
|  |  |  | 8000 | 2197 | 1331 | 729 | 512 | 125 |
| Insect |  | Sn(%) | 81.67 | 90.00 | 86.67 | 81.67 | 83.33 | 65.00 |
|  |  | Sp(%) | 99.27 | 98.90 | 98.53 | 97.07 | 96.70 | 90.84 |
|  |  | MCC | 0.86 | 0.91 | 0.88 | 0.80 | 0.81 | 0.55 |
| Invertebrate |  | Sn(%) | 64.71 | 64.71 | 61.76 | 64.71 | 58.82 | 50.00 |
|  |  | Sp(%) | 98.33 | 97.99 | 97.99 | 97.66 | 97.66 | 97.32 |
|  |  | MCC | 0.70 | 0.68 | 0.66 | 0.67 | 0.63 | 0.54 |
| Plant |  | Sn(%) | 69.05 | 83.33 | 80.95 | 76.19 | 78.57 | 59.52 |
|  |  | Sp(%) | 100.00 | 98.97 | 98.63 | 98.63 | 98.63 | 98.28 |
|  |  | MCC | 0.81 | 0.86 | 0.83 | 0.80 | 0.82 | 0.67 |
| Unclassified |  | Sn(%) | 20.00 | 35.00 | 47.50 | 40.00 | 30.00 | 10.00 |
|  |  | Sp(%) | 97.61 | 96.59 | 95.56 | 95.22 | 96.25 | 96.59 |
|  |  | MCC | 0.28 | 0.40 | 0.48 | 0.40 | 0.34 | 0.11 |
| Vertebrate |  | Sn(%) | 99.36 | 99.36 | 98.73 | 98.09 | 98.09 | 91.08 |
|  |  | Sp(%) | 68.75 | 82.95 | 85.80 | 84.66 | 81.25 | 67.61 |
|  |  | MCC | 0.71 | 0.83 | 0.88 | 0.83 | 0.80 | 0.60 |
|  | OA(%) | | 79.28 | 84.38 | 84.38 | 81.98 | 80.78 | 68.47 |

The bold values show the best results
